# Repeatability and predictive value of lactate threshold concepts in endurance sports

**DOI:** 10.1371/journal.pone.0206846

**Published:** 2018-11-14

**Authors:** Jules A. A. C. Heuberger, Pim Gal, Frederik E. Stuurman, Wouter A. S. de Muinck Keizer, Yuri Mejia Miranda, Adam F. Cohen

**Affiliations:** 1 Centre for Human Drug Research, Leiden, the Netherlands; 2 Free University of Amsterdam, Amsterdam, the Netherlands; 3 Department of Internal Medicine, Leiden University Medical Centre, Leiden, the Netherlands; University of Bourgogne France Comté, FRANCE

## Abstract

**Introduction:**

Blood lactate concentration rises exponentially during graded exercise when muscles produce more lactate than the body can remove, and the blood lactate-related thresholds are parameters based on this curve used to evaluate performance level and help athletes optimize training. Many different concepts of describing such a threshold have been published. This study aims to compare concepts for their repeatability and predictive properties of endurance performance.

**Methods:**

Forty-eight well-trained male cyclists aged 18–50 performed 5 maximal graded exercise tests each separated by two weeks. Blood lactate-related thresholds were calculated using eight different representative concepts. Repeatability of each concept was assessed using Cronbach’s alpha and intra-subject CV and predictive value with 45 minute time trial tests and a road race to the top of Mont Ventoux was evaluated using Pearson correlations.

**Results:**

Repeatability of all concepts was good to excellent (Cronbach’s alpha of 0.89–0.96), intra-subject CVs were low with 3.4–8.1%. Predictive value for performance in the time trial tests and road race showed significant correlations ranging from 0.65–0.94 and 0.53–0.76, respectively.

**Conclusion:**

All evaluated concepts performed adequate, but there were differences between concepts. One concept had both the highest repeatability and the highest predictability of cycling performance, and is therefore recommended to be used: the Dmax modified method. As an easier to apply alternative, the lactate threshold with a fixed value of 4 mmol/L could be used as it performed almost as well.

**Trial registration:**

Dutch Trial Registry NTR5643

## Introduction

The measurement of blood lactate is extensively used in sports medicine, although there is debate on how lactate affects fatigue in endurance athletes. [1] Nevertheless, the concentration of lactate in the blood relative to the exercise intensity is a relevant marker of endurance performance. [2–5] This can be visualized in a blood lactate curve (BLC) using a maximal graded exercise test (GXT): as the workload on the athlete increases over time, blood lactate concentrations (bLa) are measured at defined intervals. During high intensity contractions lactate is formed along with H^+^ in the muscles, [6] followed by an increased elimination of lactate from plasma. [7, 8] When elimination becomes saturated, bLa will start to rise when production exceeds clearance. This (exponential) rise in bLa in the BLC is of importance, as the corresponding exercise intensity is associated with endurance performance since it correlates with the transition from aerobic to anaerobic workout. [9] Since the 1960’s BLCs have been analysed trying to accurately determine a point in this curve that predicts endurance performance. Although many terms have been used for this point, in this work they will be termed lactate threshold (LT) concepts. BLCs and LT concepts can be used to assess ‘endurance fitness’ in athletes, [10] and to evaluate the effects of and to prescribe training exercises for individual athletes. [4, 5] Therefore these measures are relevant in sports medicine, both in amateur and professional sports. But as LT is based on a maximal exercise test protocol that does not directly mimic endurance exercise, finding a single point in the resulting BLC that has a strong relation to endurance performance is challenging. Moreover, determining where this single point lies in the relatively smooth curve, that is the result of a complex system of factors, can prove difficult as well. On the other hand, the more accurate method of determining maximum lactate steady state (MLSS), using several sessions with different workloads takes more time, which is the reason why an approximation of MLSS using lactate threshold concepts was developed. [11]

A previous literature review showed that there are many methods used to analyse the BLCs, with approximately 25 different concepts identified in literature to describe some form of LT. [9] These different concepts are used interchangeably throughout scientific studies and in sports and show variable repeatability and predictive value. Moreover, populations that were included in different studies often differed in training status, age and category of sport. For these reasons there is debate about these LT concepts. [9] The aim of this study is to evaluate the repeatability and predictive value of representative concepts using a large dataset of BLCs from a group of well-trained cyclists who performed multiple GXTs, time trials and an uphill road race in the setting of a clinical study.

## Materials and methods

### Study design and participants

Blood lactate curve data in this paper were generated in a previously published study. [12] Briefly, the study was a double-blind, randomized, placebo controlled, parallel, single centre trial to evaluate the effects of recombinant human erythropoietin (rHuEPO) in forty-eight healthy male cyclists aged 18 to 50. Informed consent was obtained from all individual participants included in the study. The study was approved by the Independent Ethics Committee of the Foundation Evaluation of Ethics in Biomedical Research (Stichting Beoordeling Ethiek Biomedisch Onderzoek, Assen, Netherlands). The study is registered in the Dutch Trial Registry (Nederlands Trial Register), number NTR5643. For inclusion, participants had to be well-trained, as evaluated by a maximum power-to-weight ratio during the GXT at screening that should exceed 4 W/kg. During the eleven week study duration, twenty-four participants received weekly rHuEPO injections and twenty-four received placebo injections for eight weeks. Participants had to maintain their regular training schedule during the study.

### Procedures

#### Maximal exercise tests

**Fig 1:**
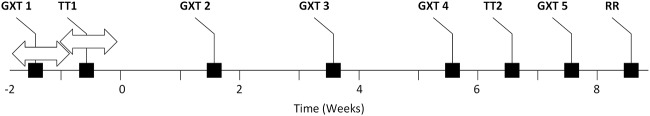
Study design. Study design showing timing of different tests. Time point 0 weeks indicates start of treatment (rHuEPO or placebo) for all participants. GXT, graded exercise test; TT, time trial test; RR, road race.

Five GXTs were performed on a Monark LC4r ergometer (COSMED, Rome, Italy) with approximately 2-week intervals between each test, see Fig 1. After a two-minute warm-up at 75 Watts, the GXT dictated an increase in pedalling resistance to 175 Watts, which increased by an additional 25 Watts every five minutes. Between 4:15 and 4:45 into each step and immediately after termination of the exercise test, blood was drawn to measure bLa. Gas exchange was measured using a Quark CPET system (COSMED, Rome, Italy) and breath-by-breath sampling technology. During the test cadence had to be maintained between 70 and 90 rpm. The test terminated when cadence could not be maintained above 70 rpm or when a participant stopped the test.

#### Lactate determination

During the GXTs blood for lactate determination was drawn via an IV cannula (Venflon 7 Pro Safety, BD, Switzerland) with a 30 cm extension set between the cannula and a three way stopcock for blood sampling in the antecubital vein. Before the first and after every sampling the stopcock and extension set were flushed with 2 mL saline. Before blood sampling 0.5 mL was withdrawn from the stopcock to remove any remaining saline. Next, 1 mL of blood was taken from the stopcock. Within ten seconds from withdrawal the blood was placed on the Lactate Pro 2 (Arkray, Kyoto, Japan) strip which was then inserted in the Lactate Pro 2 device. The same device was used throughout the whole study and was given at least 20 minutes to adjust to the room temperature before sampling.

#### Time trial tests

The time trial tests were performed twice on the same ergometer used for the GXT, with the first (TT1) 3–8 days after the first GXT and the second (TT2) one week after GXT four. Participants were instructed to produce the highest mean power output during a 45-minute period at a cadence of 70–90 rpm, attempting to mimic competitive cycling time trials. At the start of the test pedalling resistance was set at 80% of the maximal power reached during GXT1. Participants could adjust the power by indicating to increase or decrease in power by steps of 10 Watts. They were informed of the remaining time on a regular basis during the test.

#### Mont Ventoux race

Approximately one week after the last GXT participants competitively climbed the Mont Ventoux (Vaucluse département, France) via Bédoin, a climb of approximately 21.5 km with an average gradient of 7.5%. The race was preceded by a stage of 110 km in the French Provence (total elevation gain 1524 m) that was completed collectively. Racing bikes of participants were equipped with a Single Leg Power Meter SGY-PM910H2 (Pioneer Europe, Antwerp, Belgium) with Shimano Ultegra 6800 crank (Shimano, Osaka, Japan) to log power data on the bicycle during the race. Data were uploaded to the dedicated database Cyclo-Sphere.

#### Lactate threshold concepts

The BLCs from the GXTs were then used to calculate several representative LT concepts. Concepts were selected as follows: First, published concepts were retrieved from a review by Faude *et al*. [9] and by a literature research within the PubMed database. The database was searched for the search terms ‘lactate threshold’, ‘aerobic threshold’, ‘anaerobic threshold’, ‘endurance performance’ or ‘maximal lactate steady state’ or similar terms in different combinations. The references of the selected articles were searched for further relevant articles. Secondly, retrieved concepts were divided into seven different categories, see S1 Table. A few retrieved concepts could not be implemented, reasons being lacking lactate concentrations in the recovery phase after exercise and no availability of the full text article describing the method of the concept despite various efforts obtaining it. (S1 Table, listed under “not selected categories”). From each remaining category, concepts that were representative and were used frequently in other research were selected. If there were multiple concepts in one category that were commonly used and fundamentally different in methodology, more than one concept of that category was included in the analysis. Selecting multiple commonly used, but very similar concepts from one category was not deemed useful for the purpose of this study. This resulted in a final selection of eight concepts from the five implementable categories for analysis in our study.

#### Implementation of lactate threshold concepts

**Fig 2:**
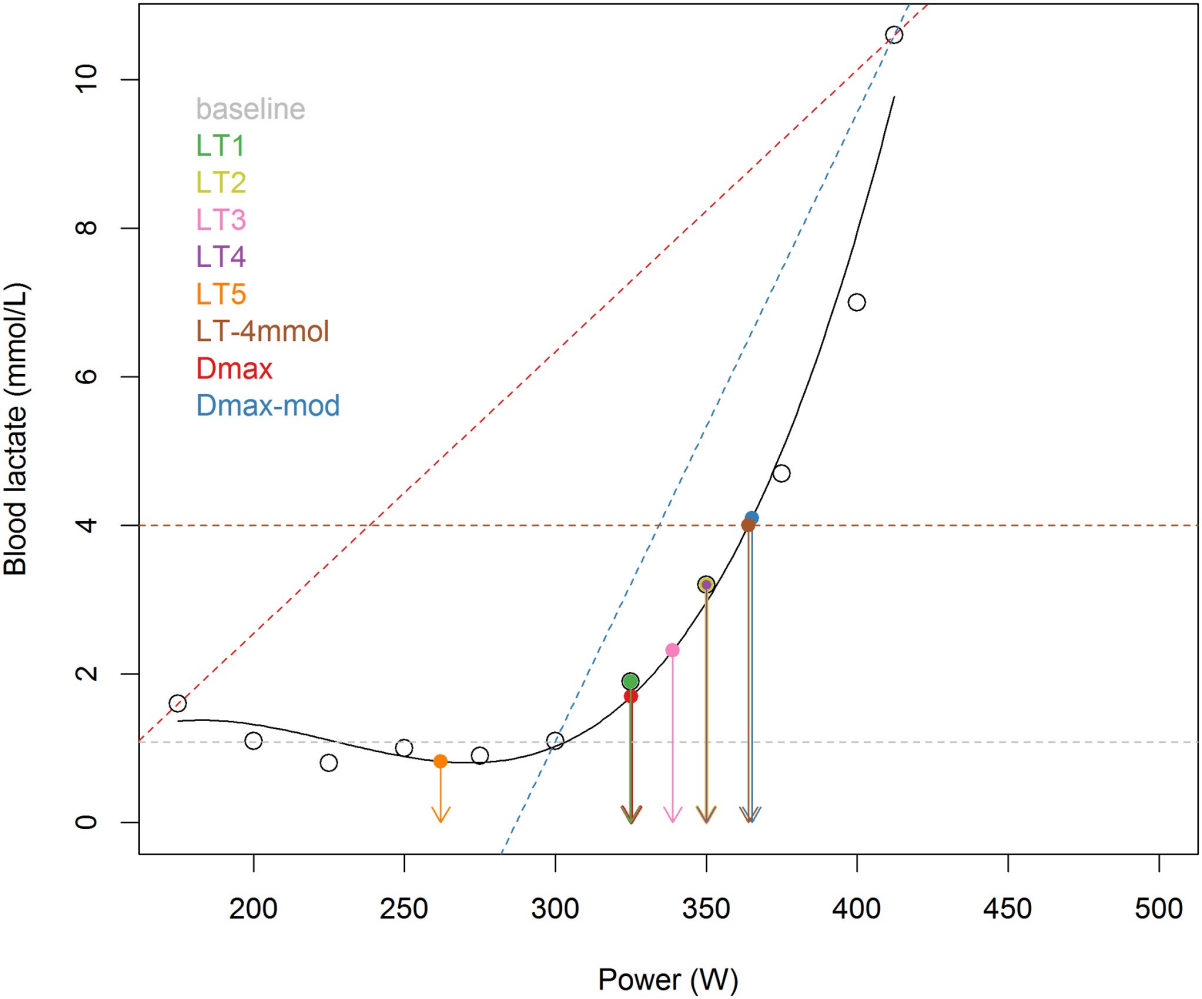
Graphical representation of lactate threshold concepts. Example of a blood lactate curve with the location of the different lactate threshold concepts for this particular curve. Open circles: observed blood lactate values at each exercise intensity; Black curve: third-order polynomial; Grey dashed line: baseline; Green circle and arrow: LT1, observer-determined first rise in blood lactate; Yellow circle and arrow: LT2, first observed blood lactate value more than 1 mmol/L above baseline; Pink circle and arrow: LT3, minimum lactate equivalent (blood lactate divided by power) plus 1.5 mmol/L; Purple circle and arrow: LT4, first blood lactate value that shows an increase of at least 1 mmol/L; Orange circle and arrow: LT5, minimum lactate equivalent (blood lactate divided by VO2); Brown circle and arrow and dashed line: LT-4mmol, value at 4 mmol/L; Red circle and arrow and dashed line: Dmax, value with the maximum perpendicular distance to the polynomial from the dashed line; Blue circle and arrow and dashed line: Dmax-mod, value with the maximum perpendicular distance to the polynomial from the dashed line.

All selected concepts were implemented according to the articles that described the concept (S1 Table). When exact reproduction of the method was not feasible due to the use of different parameters (e.g. running velocity was used), we approximated the description as close as possible (e.g. we used power output). For concepts that required data fitting of the blood lactate curve a third-order polynomial was chosen, based on the shape of the blood lactate curve data and given that it is a proven method, although there is no generally accepted method for data fitting. [9] An example of a blood lactate curve with a depiction of all lactate threshold concepts is shown in Fig 2.

#### LT1

Similar to what Tanaka described [13] we plotted bLa (mmol/L) *versus* power (W). Three authors (JH, WdMK and PG) were asked to independently select the first point in the BLC that marks a substantial increase above resting level. LT1 was defined as the power value corresponding to the point selected by at least two researchers, or in cases without consensus, the three researchers discussed until consensus was reached.

#### LT2

Coyle *et al*. [14] determined LT as 1 mmol/L above a visually determined baseline in the BLC. We took the lactate measurement chosen as LT1 and calculated the mean of the measurements preceding this point to create an average baseline value. The power value belonging to the first measured lactate value after baseline that supersedes the baseline value plus 1 mmol/L was considered LT2.

#### LT3

As Dickhuth *et al*., [15] we determined the minimum lactate equivalent (the lowest value when bLa is divided by work intensity) using third-order polynomial fitting and added 1.5 mmol/L to the corresponding bLa, termed individual anaerobic threshold in the paper, to find the power value on the fitted polynomial of the BLC and termed it LT3.

#### LT4

As described by Amann *et al*., [16] we calculated the first rise of 1 mmol/L or more between two bLa measurements where the next rise was similar or larger than 1 mmol/L. The measurement that preceded this first increase was considered LT4.

#### LT5

Based on the method described by Dickhuth *et al*., [17] we divided bLa (mmol/L) by the 30 second average VO_2_ (mL/min/kg) and plotted it against power. These values were interpolated with a third-order polynomial and the power value at the lowest point in this curve was considered LT5.

#### LT-4mmol

A widely used concept is the LT-4mmol method, as described for example by Sjodin *et al*. [18] The power in the interpolated third-order polynomial BLC that corresponds to a bLa of 4 mmol/L was considered LT-4mmol.

#### Dmax and Dmax modified

Similar to the method proposed by Cheng *et al*., [19] we plotted bLa versus power, interpolated with a third-order polynomial and plotted a line from the first measurement to the last measurement. The point in the interpolated BLC that has the maximum perpendicular distance with that line was considered Dmax. A modified version as described by Bishop *et al*., [20] uses the measurement that precedes an increase of at least 0.4 mmol/L instead of the first bLa measurement to draw the line to the last measurement, which is termed Dmax modified (Dmax-mod).

### Data management

Data was stored in a validated database system (Promasys, Omnicomm Inc., Fort Lauderdale, USA) and checked for accuracy and completeness. Blinded data review before code-breaking and analysis was performed according to a standard procedure at our unit. This included evaluating whether the GXT was performed to maximal ability, which was based on power, VO_2_ and bLa values and report by the subject.

### Statistical analysis

We used statistical software R version 3.4.0 [21] to plot measurements, calculate the third-order polynomial that best fits the data using polynomial regression with the R-function lm(y~poly(3)), implement the LT concepts and perform the statistical testing. R was used with the following packages: dplyr 0.5.0, [22] psych 1.7.5, [23] tidyr 0.6.3. [24] Data of all subjects enrolled in the study were used in the analysis.

#### Repeatability

To measure repeatability we determined the weighted intra-subject coefficient of variation (CV) and the Cronbach’s alpha based on the five GXT results for each LT concept. Weighted intra-subject CV was calculated correcting for missing values (CV based on the sum of the variance per subject multiplied by the amount of measurements, divided by the total amount of measurements). Both the weighted intra-subject CV and Cronbach’s alpha were calculated only using data from participants receiving placebo, as there might have been longitudinal effects of rHuEPO treatment on the GXTs.

#### Predictive properties

For the predictive properties we calculated the Pearson correlation between each LT concept and the mean power of the corresponding relevant endurance parameter. The LT concept from the GXT closest in time to the endurance tests TT1 and TT2 and road race (see Fig 1), namely GXT 1, 4 and 5 respectively, were used for correlations between the LT concept and corresponding average power output. In addition, the difference between each measurement pair was calculated and averaged to create the mean difference between the LT concept and endurance test power. This value indicates how the power at the LT concept translates to average endurance power in a time trial or race. For these Pearson correlation and mean difference analyses both subjects receiving rHuEPO and placebo were included. This was done as LT concepts are designed to be a predictive parameter for endurance exercise, which should be irrespective of a subject being treated with rHuEPO or not. In addition, given that the measurements of each pair are at most a week apart, no changes in the LT concept or endurance performance are expected due to rHuEPO. Moreover, GXT1 and TT1 were performed before starting the treatment period, and no rHuEPO administrations took place between GXT5 and the race. For these analyses therefore no treatment effect was expected and pooling was considered appropriate.

## Results

**Fig 3:**
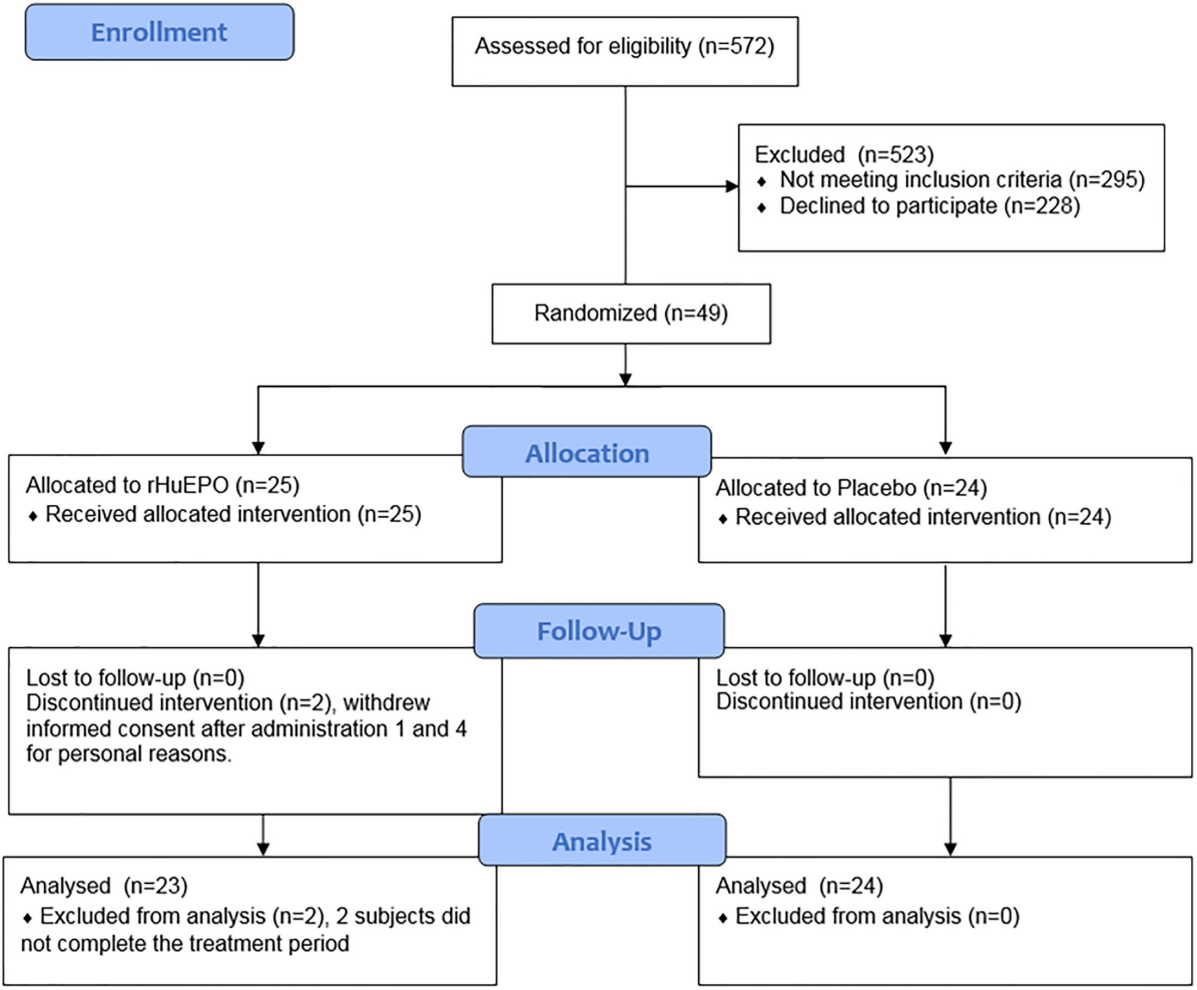
CONSORT flowchart.

In total 49 subjects entered the study, of which 47 were completers (Fig 3); one subject dropped out after having performed the first GXT and time trial test and was replaced. One other subject dropped out after completing two GXTs and one time trial test and was not replaced. Subject characteristics can be found in Table 1. Of the remaining 238 planned GXTs, five were not performed due to illness or injury. An additional 22 were excluded from analysis, five due to having less than four bLa samples for the GXT, most others due to the fact that subjects indicated having physical problems (e.g. illness/injury, sore legs from recent exercise) potentially affecting test results, leaving 211 GXTs (of which 109 from placebo subjects) with analysable lactate threshold data. A total of 96 time trial tests were performed and used in the analysis, and power data of 37 subjects was available for the road race. Out of the 47 subjects that completed the study, three could not participate in the road race, four did not reach the finish line due to exhaustion, and three did not have a power meter on their bike, therefore lacking power data for the road race.

**Table 1:** Subject disposition.

	All subjects	Placebo subjects
N	48	24
Age (years)	33.6 (20.0–50.0)	33.8 (20.0–50.0)
Weight (kg)	76.9 (9.0; 59.2–95.6)	76.9 (8.9; 59.2–95.6)
Height (cm)	186 (7.3; 172–203)	186 (6.7; 174–203)
Maximal Power output per kg (W/kg)	4.36 (4.03–5.18)	4.36 (4.03–4.94)
VO_2,max_ (mL/min/kg)	55.7 (4.6; 45.3–67.5)	56.0 (4.1; 47.0–62.8)

Values are presented as mean (standard deviation (SD) where appropriate; range where). VO_2,max_: maximal oxygen consumption.

### Lactate threshold concepts and endurance

All eight LT concepts were successfully implemented on the GXT data; for LT1 which was determined visually by three researchers, a unanimous decision about the lactate threshold was reached in 56.8% of the tests, in 40.0% of the cases two out of three researchers agreed and there was originally no consensus in 3.2% of the tests. Several concepts were based on the third-order polynomial data fitting, mean r-squared values of all individual curves were 0.978 (SD = 0.032, range 0.716–1.000). Mean values for each LT concept of the placebo group can be found in Table 2. Mean (SD) power output for TT1 was 268 W (28 W) in the placebo group and 271 W (29 W) in the rHuEPO group, and estimated mean for TT2 was 277 W and 283 W for the placebo and rHuEPO groups respectively. Estimated mean power during RR were 266 W and 257 W for the two groups, during a mean race time of 1 h 37 min 45 s (SD = 12 min 40 s) and 1 h 38 min 23 s (SD = 14 min 9 s), respectively.

**Table 2:** Mean lactate threshold concept power output.

GXT number	LT1 (W)	LT2 (W)	LT3 (W)	LT4 (W)	LT5 (W)	LT-4mmol (W)	Dmax (W)	Dmax-mod (W)
**1**	283.3 (29.9; 225–350)	292.9 (37.2; 250–375)	286.1 (32.9; 219–352)	275.0 (41.1; 200–350)	225.0 (31.2; 175–283)	301.8 (41.0; 222–381)	275.7 (24.6; 222–323)	299.5 (35.3; 225–367)
**2**	283.0 (22.3; 225–375)	293.2 (31.0; 250–375)	288.7 (29.2; 231–373)	276.1 (34.0; 175–375)	231.8 (25.7; 175–300)	305.0 (33.0; 234–389)	280.0 (23.6; 233–339)	301.2 (28.7; 237–369)
**3**	281.0 (29.5; 225–400)	290.5 (27.9; 250–400)	285.7 (26.3; 240–390)	272.6 (33.5; 225–375)	224.5 (29.5; 175–318)	300.8 (28.9; 253–411)	278.7 (20.9; 250–343)	297.5 (29.6; 257–413)
**4**	283.7 (35.8; 225–400)	292.4 (38.0; 225–425)	291.6 (29.1; 240–392)	272.8 (36.9; 200–400)	229.8 (29.5; 175–323)	307.0 (34.2; 249–415)	284.0 (22.7; 232–338)	308.7 (35.6; 251–396)
**5**	278.3 (28.5; 225–325)	290.2 (37.5; 225–350)	285.0 (31.6; 216–339)	271.7 (37.9; 200–350)	230.4 (30.5; 175–274)	297.2 (38.9; 204–364)	280.3 (20.3; 245–325)	298.9 (29.2; 253–365)
**Overall**	282.1 (5.7%)	292.2 (5.0%)	287.7 (3.6%)	274.1 (5.6%)	228.4 (8.1%)	302.7 (3.8%)	280.0 (3.4%)	301.5 (4.3%)

Weighted mean power output (SD; range) for the placebo group at every exercise test. Overall combined (based on 109 GXTs) for each lactate threshold concept (CV). CV is weighted intra-subject CV.

### Repeatability

The overall intra-subject CV of each LT concept is indicated in Table 2, and shows some minor differences between concepts, with LT3, LT-4mmol, Dmax and Dmax-mod having CVs < 5% and LT5 having the highest intra-subject CV with 8.1%. The Cronbach’s alpha values for all LT concepts in the placebo group are between 0.89 and 0.97 and although 95% CIs largely overlap, the same four concepts as observed for intra-subject CVs perform best with Cronbach’s alpha values >0.95 (Table 3).

**Table 3:** Cronbach’s alpha for each lactate threshold concept.

Lactate threshold concept	Cronbach’s alpha	Lower 95% CI	Upper 95% CI
**LT1**	0.91	0.85	0.96
**LT2**	0.95	0.92	0.98
**LT3**	0.97	0.94	0.99
**LT4**	0.94	0.91	0.98
**LT5**	0.89	0.82	0.96
**LT 4_mmol**	0.96	0.94	0.99
**Dmax**	0.96	0.93	0.98
**Dmax-mod**	0.96	0.94	0.98

Cronbach’s alpha for the placebo group for each lactate threshold concept with 95% confidence interval (CI).

### Predictive properties

**Fig 4:**
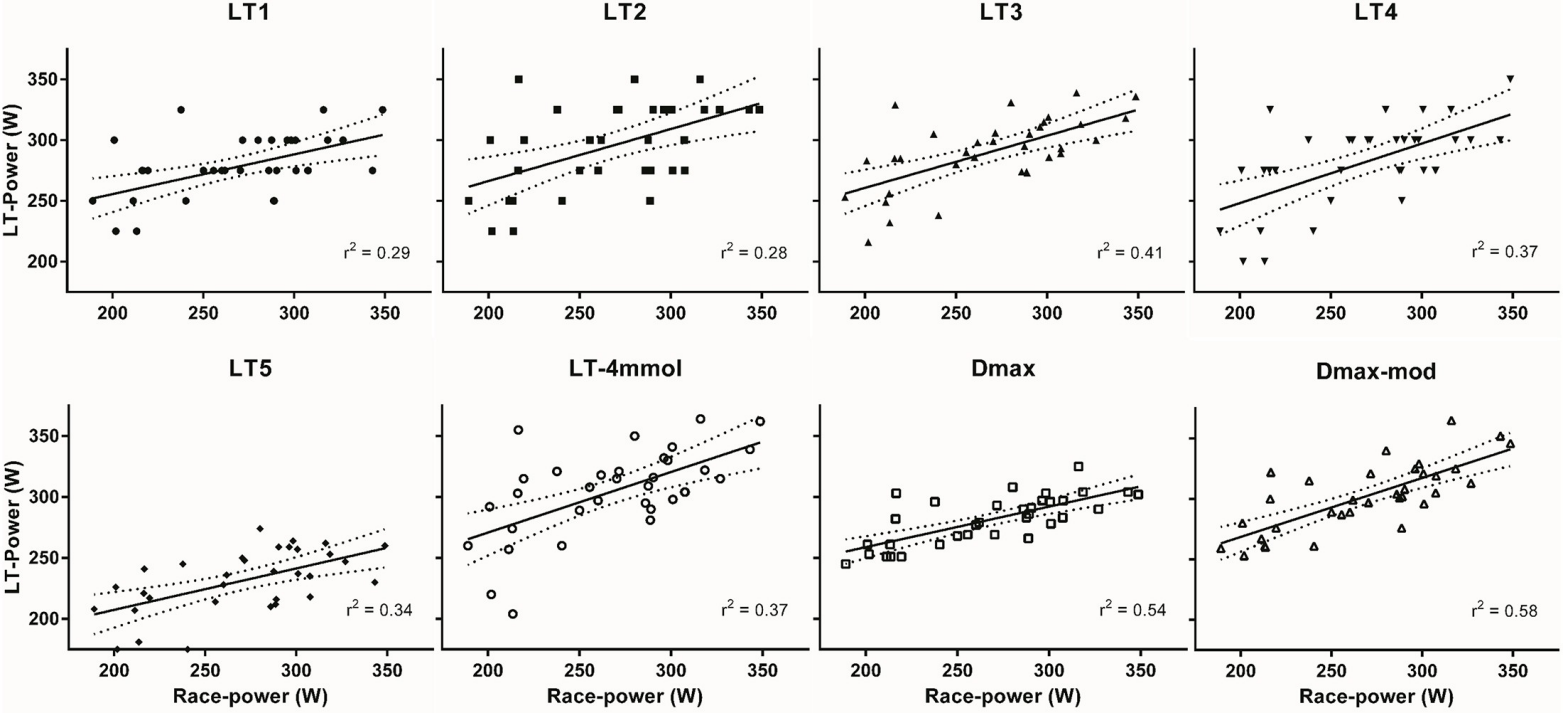
Linear regression lactate threshold concept power and average race power. Linear regression of lactate threshold power and average race power per LT concept for all subjects depicting linear regression line (solid line) and 95% confidence interval (dotted lines). r^2^: R-squared or coefficient of determination is the proportion of the variance in the dependent variable that is predictable from the independent variable.

Pearson correlation coefficients and the mean difference between each correlation pair are listed in Table 4. All correlations are highly significant (p<0.0002), indicating the null hypothesis that the correlation is equal to zero can be rejected. The strength of the relationship differs for different concepts. Correlation with TT1 was very strong for Dmax-mod and strong for all other concepts except LT5, which showed a moderate correlation. Correlation with TT2 was strong for all concepts except LT5, which showed a moderate correlation. Correlation with RR was strong for Dmax and Dmax-mod, and moderate for all other concepts. Dmax-mod has the highest correlation with time trial test 1 (r = 0.94), LT-4mmol with time trial test 2 (r = 0.85) and Dmax-mod with road race power (r = 0.76). The mean difference with the endurance parameters differs substantially between concepts, ranging from the lactate threshold on average being up to 45.3 W lower than the related endurance parameter for LT5 to 36.6 W higher for LT-4mmol. Linear regression between each LT concept and average race power, including accompanying r^2^ values, is plotted in Fig 4.

**Table 4:** Predictive value of lactate threshold concepts.

Lactate threshold concept	Pearson correlation			Mean difference (SD)		
	*TT1*	*TT2*	*RR*	*TT1*	*TT2*	*RR*
	*n = 42*	*n = 46*^a^	*n = 34*^b^	*n = 42*	*n = 46*^a^	*n = 34*^b^
**LT1**	0.78	0.74	0.54	-11.3 (18.3)	-8.7 (22.1)	-9.8 (37.2)
**LT2**	0.87	0.80	0.53	-23.2 (16.0)	-18.5 (19.3)	-27.4 (39.3)
**LT3**	0.88	0.84	0.64	-16.2 (14.3)	-16.1 (18.1)	-21.9 (33.8)
**LT4**	0.78	0.82	0.61	-3.5 (23.7)	-0.6 (26.2)	-13.5 (36.1)
**LT5**	0.67	0.65	0.58	43.7 (21.7)	45.3 (23.5)	39.1 (35.9)
**LT-4mmol**	0.88	0.85	0.61	-31.7 (19.4)	-32.3 (23.0)	-36.6 (36.1)
**Dmax**	0.89	0.82	0.73	-4.4 (12.1)	-3.8 (15.8)	-13.4 (32.4)
**Dmax-mod**	0.94	0.84	0.76	-27.3 (11.8)	-29.9 (16.6)	-33.7 (29.1)

Pearson correlation between each lactate threshold concept in GXT 1 and time trial test 1 (TT1), GXT 4 and time trial test 2 (TT2) and GXT 5 and average road race (RR) power for all subjects combined. All correlations are significant (p<0.0002). To determine potential differences in power output between the LT concept and time trial power or race power, mean difference (SD) between each measurement pair is calculated. Negative values indicate lactate threshold power is higher than exercise test average power.

^a^ For LT5 n = 44;

^b^ for LT5 n = 32.

## Discussion

All LT concepts that were included in this analysis performed good on repeatability and reasonable to good on predicting a lab-based time trial and a real-life road race. Nevertheless, this study identified several LT concepts that outperformed the others in the setting of this trial. The best method being Dmax-mod, but Dmax, LT-4mmol and LT3 performed well too.

### Methodology

The design of the exercise protocol, for example stage duration, is known to impact blood lactate curves. [25, 26] We selected an exercise protocol with five minute stages and 25 W increments because it takes 3–4 minutes for the body to reach steady state and lactate accompanying that effort level can be measured accurately. [27] In addition, longer protocols may be more sensitive to performance changes. [25] As described in more detail elsewhere, [12] GXT results show our subjects were well-trained with maximal power output and VO2 max values comparable to elite cyclists and triathletes when using longer exercise protocols. [28, 29] All evaluated concepts were applied to data from the same exercise tests, with the same sampling and assay method, and the same fitting procedure was used for those applicable concepts. As a result such factors could not have affected the comparison between concepts within this study. The current study was designed in that way to give the most accurate estimate of performance parameters and its controlled set-up seems to be the most robust and valuable way to determine differences between concepts. Nevertheless, when any of these factors are changed (e.g. using a different exercise test protocol) it is possible the outcomes might not translate perfectly. With regards to data fitting, the third-order polynomial in the applicable concepts performed well given the high mean r-squared values observed.

### Selection of concepts

After inspection of all identified lactate concepts, it became clear that there were similarities between quite some of them. For this reason, the concepts were grouped into categories, and a selection was made of concepts to be analysed to have at least one representative per category and thereby ensuring that results from this study would be informative for all regularly used lactate concepts. The selection includes concepts such as a fixed lactate value (usually at 4 mmol/L) and the visually determined LT concepts that have been used since the conception of the LT, and more recent concepts such as LT3, LT4 and Dmax and Dmax-mod. [9]

### Mean threshold

The mean power output (Table 2) is relatively constant over time for each concept. These results confirm there was no placebo-effect on any of the LT concepts, although such an impact would already theoretically be improbable. What can also be seen is that not all concepts seem to identify the same point in the blood lactate curve: LT5 gives the lowest estimate of LT (228.4 W), much lower than other concepts (274.1–302.7 W). LT-4mmol and Dmax-mod have the highest estimates (302.7 and 301.5 W), indicating these concepts identify different intensities of performance and have different physiological meanings. Applying the terminology as described in Faude *et al*, [9] based on mean threshold and mean difference with TT and RR (Table 4), some concepts seem to be more related to the aerobic threshold (LT5), others to the aerobic/anaerobic transition (e.g. LT1, LT4, Dmax) or the anaerobic threshold (LT-4mmol, Dmax-mod).

### Repeatability

Intra-subject CV’s over all five measurements were low (3.4–8.1%) and Cronbach’s alphas high (0.89–0.97), indicating repeatability of all concepts over the study period of approximately 8 weeks was good. This corresponds well to previous findings of repeatability for power or speed at different lactate concepts, both in terms of CV, determined at 1.3–5.9% in a meta-analytic review, [30] and in terms of Pearson correlations 0.88–0.96 [31, 32] or ICC 0.98–0.99. [33] One study applied different LT concepts to the same dataset from two exercise tests and showed that intra-subject CV’s and correlation was good for LT2, LT-4mmol and a concept similar to LT4 (CV 3–4% and r ≥ 0.85), but not for Dmax (10.3% and 0.57). [34] Our data, based on more subjects (24 versus 14) and more measurements per subject (5 versus 2), disputes this relatively poor repeatability for Dmax. However, our study does show differences between concepts, with LT3, LT-4mmol, Dmax and Dmax-mod having the lowest intra-subject CV (<5%) and the highest Cronbach’s alpha (>0.95).

### Correlation with performance

As we have established that CV and repeatability for all LT concepts was good, the most relevant question is whether these concepts correlate to actual endurance performance. As previously indicated, it is highly unlikely that the rHuEPO treatment impacted this particular correlation analysis. When analysing the groups separately, some differences in correlation coefficients could be observed between the two groups (data not shown), but these differences were already present for the correlation between GXT1 and TT1 when treatment had not yet started, indicating that this was not due to rHuEPO treatment. Because combining all subjects generates more informative and robust results being based on a bigger population, pooling the groups was considered justified.

Data in Table 4 show that for all concepts correlations with time trial tests were higher compared to the road race (based on all subjects median of all concepts r = 0.875 for TT1 and 0.82 for TT2, *versus* 0.61 for RR). This is most likely partly due to additional variability in the road race due to the circumstances (e.g. weather, uphill racing with changes in steepness over the course, and differences in race duration (range 72–126 minutes)). Possibly there was also a minor impact of using different equipment for power measurement during the RR, as it was not measured on the ergometer but on the subjects’ bike. What can also be seen is that correlation of the LT concepts with TT1 in general is slightly higher than with TT2. More importantly however, correlation for both time trials show that the ranking among different concepts is very similar, confirming the results are robust. It seems that in general, using a technique of interpolation for the BLC has superior performance, as LT concepts that were based on the third-order polynomial derived from the individual lactate concentration measurements (LT3, LT5, LT-4mmol, Dmax, Dmax-mod) performed better than the ones that used actual measured bLa values without interpolation (LT1, LT2 and LT4), with the exception of LT5. This poor performance of LT5 is most likely due to the fact that it is conceptually different from the other concepts; it is the power at the minimum lactate equivalent, in this case the lowest value for the lactate-VO2 ratio. In contrast, LT3 also uses a form of the minimum lactate equivalent, but it adds 1.5 mmol/L to this value. As can be seen in Table 2 and Fig 2, this leads to LT5 on average determining a point even before the first rise in lactate concentration as determined by LT1. This concept therefore relates to much lower (aerobic) work intensities than the other concepts. Additionally it is less repeatable (see Table 3). From all tested concepts LT5 correlates least with 45 min TT performance, but for the longer RR performance relative to the other concepts it performs somewhat better than for TT. This could mean that is this concept is more related to long-term exercise efforts.

Many studies previously evaluated correlations of LT concepts with endurance performance, of which most used running performance. An overview of these studies by Faude *et al* shows a median r = 0.84–0.92 for several different LT concepts for endurance distances (>5km), [9] comparable to our results. There are fewer studies that have compared LT concepts and their correlation with different types of cycling endurance performance, [16, 20, 26, 35–38] but correlation with endurance performances (30–90 minutes) for each concept seem to vary between these studies, see Table 5. In addition, the comparison between concepts within these studies shows varying conclusions about which is the best concept. This could partially be due to differences between studies, for example study populations differ (mean VO2max ranges from 48 to 68 mL/kg/min, and some studying female, others male cyclists and/or triathletes). However, they are more or less as heterogeneous as our population with an SD of 4–8 mL/kg/min on VO2max. The applied exercise protocols all used long stages similar to ours (3–5 minutes), although the increases in workload differ (20-50W). Finally, correlation to endurance exercise was based on time trials that lasted between approximately 30 to 90 minutes (our TT of 45 min at the lower end and RR of on average 98 min at the higher end), a difference that might impact the correlation to different LT concepts. Nevertheless, taking these differences into account, comparison is possible, albeit with some caution. Moreover, a robust and valid LT concept should perform well in any of these datasets. What can be observed is that all these concepts except LT1, Dmax and Dmax-mod have shown correlations below 0.75, and that in all four direct comparisons that evaluated both Dmax and LT-4mmol, Dmax showed a higher correlation. This latter finding could be due to the fact that LT-4mmol is less robust to changes in settings such as exercise protocol duration, sampling site and lactate analyser because of its fixed nature. Our study expands on this information, and compared to previous studies as reviewed in Table 5, is based on approximately 2–4 times more subjects, therefore allowing for more robust conclusions. This is especially true since our population is a heterogeneous well-trained, and therefore relevant, group (range maximal power output at baseline 256–425 W). Similar to what can be extracted from the literature, our study too shows that Dmax and Dmax-mod have highest correlations with time trial performance, although LT-4mmol and LT3 show a similarly high correlation in our study. For the correlation with RR, there are slightly larger differences between concepts. Correlation is highest for Dmax and Dmax-mod, mainly because for the other concepts correlation for a few subjects is very poor, as visualized in Fig 4 (e.g. for LT-4mmol). These findings combined, we conclude that Dmax, and even more so Dmax-mod, have the best correlation with endurance performance. One recent study evaluated correlation between MLSS, which could be considered to be the gold standard for the physiological endurance threshold, and different LT concepts generated from GXTs with different protocol durations. [26] This study concluded that for a GXT with 4-minute steps (most similar to our GXT), correlation was high for many of the concepts, but validity was highest for LT-2.5mmol, Dmax-mod, and two modified versions of Dmax-mod. In contrast, LT2, LT-4mmol and Dmax showed much higher mean differences with MLSS and therefore were designated as invalid estimates of MLSS. Combining these findings with our own results, Dmax-mod determined in a GXT with approximately 5-minute stages is both a valid estimate of MLSS and has a high correlation with actual endurance performance.

**Table 5:** Reported correlations between LT concepts and endurance performance.

Correlation reported in publication	Lactate threshold concept						
	LT1	LT2	LT4	LT-4mmol	Dmax	Dmax-mod	LTlog
Amann [16]	-	0.72	0.59	0.60	-	-	-
Bentley [37]	-	-	-	0.54	0.77	-	0.91
Bishop [20]	0.81	-	0.61	0.81	0.84	0.83	0.69
Borszcz [35]	-	0.31	-	0.56	0.75	-	-
McNaughton [38]	-	-	-	0.90	0.91	-	0.86
Nichols [36]	-	-	0.88	0.67	-	-	-

Literature data for LT concepts and correlation with 30–90 minute-during performances. LTlog: the power output at which bLa starts to increase when log(bLa) is plotted against log(power output).

### Absolute power difference

The mean difference of each concept with the endurance parameter gives an indication of how the absolute power of the LT concept corresponds to the average power produced during TT and RR. On average, power is higher compared to the endurance test for each concept (except the poorest performing concept LT5). This difference in power between LT concepts and endurance test is possibly due to having to sustain the power for a much longer time during the endurance tests, needing a systematic lower power in order to cope with the effort. Interestingly, Dmax-mod and LT-4mmol, concepts that show among the highest correlations, have the largest difference in absolute power (approximately 30 W). Given the high correlation with performance this should not disqualify these concepts, but one should take into account that there is a systematic difference with endurance performance of approximately 30 W.

## Conclusions

LT concepts are correlated with endurance performance, but a review showed that many different concepts are used in literature, which is undesirable. [9] Also for cycling performance, there is no consensus on which LT concept should be applied and results vary highly. [16, 20, 35–38] In this study we compared eight different representative LT concepts on the same large cycling performance dataset to evaluate repeatability and predictive properties. All concepts showed high repeatability, and correlated with endurance performance. However, LT3, LT-4mmol, Dmax and Dmax-mod showed the best repeatability, and had the highest correlation with time trial performance. As correlation with performance was consistently high for Dmax and Dmax-mod, also with the uphill road race, the latter performing slightly better on each criterion, and because Dmax-mod was previously shown to be a valid estimate of MLSS, we would recommend using Dmax-mod when analyzing the blood lactate curve.

## Supporting information

S1 Table Lactate threshold concept categories. (DOCX)

S1 Protocol Study protocol CHDR1514. (PDF)

## References

[1] Cairns SP. Lactic acid and exercise performance: culprit or friend? Sports Med. 2006;36(4):279–91. 10.2165/00007256-200636040-00001 16573355

[2] Atkinson G, Davison R, Jeukendrup A, Passfield L. Science and cycling: current knowledge and future directions for research. J Sports Sci. 2003;21(9):767–87. 10.1080/0264041031000102097 14579871

[3] Kindermann W, Simon G, Keul J. The significance of the aerobic-anaerobic transition for the determination of work load intensities during endurance training. Eur J Appl Physiol Occup Physiol. 1979;42(1):25–34. 499194 10.1007/BF00421101

[4] Londeree BR. Effect of training on lactate/ventilatory thresholds: a meta-analysis. Med Sci Sports Exerc. 1997;29(6):837–43. 9219214 10.1097/00005768-199706000-00016

[5] Antonutto G, Di Prampero PE. The concept of lactate threshold. A short review. J Sports Med Phys Fitness. 1995;35(1):6–12. 7474995

[6] Robergs RA, Ghiasvand F, Parker D. Biochemistry of exercise-induced metabolic acidosis. Am J Physiol Regul Integr Comp Physiol. 2004;287(3):R502–16. 10.1152/ajpregu.00114.2004 15308499

[7] MacRae HS, Dennis SC, Bosch AN, Noakes TD. Effects of training on lactate production and removal during progressive exercise in humans. J Appl Physiol (1985). 1992;72(5):1649–56. 1601768 10.1152/jappl.1992.72.5.1649

[8] Stanley WC, Gertz EW, Wisneski JA, Neese RA, Morris DL, Brooks GA. Lactate extraction during net lactate release in legs of humans during exercise. J Appl Physiol (1985). 1986;60(4):1116–20. 3084443 10.1152/jappl.1986.60.4.1116

[9] Faude O, Kindermann W, Meyer T. Lactate threshold concepts: how valid are they? Sports Med. 2009;39(6):469–90. 10.2165/00007256-200939060-00003 19453206

[10] Coyle EF, Coggan AR, Hopper MK, Walters TJ. Determinants of endurance in well-trained cyclists. J Appl Physiol (1985). 1988;64(6):2622–30. 3403447 10.1152/jappl.1988.64.6.2622

[11] Billat VL, Sirvent P, Py G, Koralsztein JP, Mercier J. The concept of maximal lactate steady state: a bridge between biochemistry, physiology and sport science. Sports Med. 2003;33(6):407–26. 10.2165/00007256-200333060-00003 12744715

[12] Heuberger J, Rotmans JI, Gal P, Stuurman FE, van’t Westende J, Post TE, et al Effects of erythropoietin on cycling performance of well trained cyclists: a double-blind, randomised, placebo-controlled trial. Lancet Haematol. 2017;4(8):e374–e86. 10.1016/S2352-3026(17)30105-9 28669689

[13] Tanaka H. Predicting running velocity at blood lactate threshold from running performance tests in adolescent boys. Eur J Appl Physiol Occup Physiol. 1986;55(4):344–8. 3758032 10.1007/BF00422731

[14] Coyle EF, Martin WH, Ehsani AA, Hagberg JM, Bloomfield SA, Sinacore DR, et al Blood lactate threshold in some well-trained ischemic heart disease patients. J Appl Physiol Respir Environ Exerc Physiol. 1983;54(1):18–23. 10.1152/jappl.1983.54.1.18 6826403

[15] Dickhuth H-H, Yin L, Niess A, Rocker K, Mayer F, Heitkamp HC, et al Ventilatory, lactate-derived and catecholamine thresholds during incremental treadmill running: relationship and reproducibility. Int J Sports Med. 1999;20(2):122–7. 10.1055/s-2007-971105 10190774

[16] Amann M, Subudhi AW, Foster C. Predictive validity of ventilatory and lactate thresholds for cycling time trial performance. Scand J Med Sci Sports. 2006;16(1):27–34. 10.1111/j.1600-0838.2004.00424.x 16430678

[17] Dickhuth H.-H.; Huonker M. MT, Drexler H., Berg A., Keul J. Individual anaerobic threshold for evaluation of competitive athletes and patients with left ventricular dysfunctions. Advances in ergometry. 1991.

[18] Sjodin B, Jacobs I. Onset of blood lactate accumulation and marathon running performance. Int J Sports Med. 1981;2(1):23–6. 10.1055/s-2008-1034579 7333732

[19] Cheng B, Kuipers H, Snyder AC, Keizer HA, Jeukendrup A, Hesselink M. A new approach for the determination of ventilatory and lactate thresholds. Int J Sports Med. 1992;13(7):518–22. 10.1055/s-2007-1021309 1459746

[20] Bishop D, Jenkins DG, Mackinnon LT. The relationship between plasma lactate parameters, Wpeak and 1-h cycling performance in women. Med Sci Sports Exerc. 1998;30(8):1270–5. 9710868 10.1097/00005768-199808000-00014

[21] Chambers J. Project R The R Project for Statistical Computing [3.4.0:[https://www.r-project.org/.

[22] Hadley Wickham RF, Lionel Henry, Kirill Müller. dplyr: A Grammar of Data Manipulation dplyr: A Grammar of Data Manipulation2017 [https://cran.r-project.org/web/packages/dplyr/.

[23] Revelle W. psych: Procedures for Psychological, Psychometric, and Personality Research psych: Procedures for Psychological, Psychometric, and Personality Research2017 [https://cran.r-project.org/web/packages/psych/.

[24] Hadley Wickham LH. tidyr: Easily Tidy Data with ’spread()’ and ’gather()’ Functions tidyr: Easily Tidy Data with ’spread()’ and ’gather()’ Functions2017 [https://cran.r-project.org/web/packages/tidyr/.

[25] Bentley DJ, Newell J, Bishop D. Incremental exercise test design and analysis: implications for performance diagnostics in endurance athletes. Sports Med. 2007;37(7):575–86. 10.2165/00007256-200737070-00002 17595153

[26] Jamnick NA, Botella J, Pyne DB, Bishop DJ. Manipulating graded exercise test variables affects the validity of the lactate threshold and [Formula: see text]. PLoS One. 2018;13(7):e0199794 10.1371/journal.pone.0199794 30059543 PMC6066218

[27] Thoden JS. Testing aerobic power In: MacDougall JD, Wenger HA, Green HJ, editors. Physiological testing of the high-performance athlete. Champaign: Human Kinetics; 1991 p. 107–74.

[28] San Millan I, Bing K, Brill C, Hill JC, Miller LE. Randomized controlled trial of Micro-Mobile Compression(R) on lactate clearance and subsequent exercise performance in elite male cyclists. Open Access J Sports Med. 2013;4:221–7. 10.2147/OAJSM.S51956 24379728 PMC3871408

[29] Bentley DJ, McNaughton LR. Comparison of W(peak), VO2(peak) and the ventilation threshold from two different incremental exercise tests: relationship to endurance performance. J Sci Med Sport. 2003;6(4):422–35. 14723392 10.1016/s1440-2440(03)80268-2

[30] Hopkins WG, Schabort EJ, Hawley JA. Reliability of power in physical performance tests. Sports Med. 2001;31(3):211–34. 10.2165/00007256-200131030-00005 11286357

[31] Weltman A, Snead D, Stein P, Seip R, Schurrer R, Rutt R, et al Reliability and validity of a continuous incremental treadmill protocol for the determination of lactate threshold, fixed blood lactate concentrations, and VO2max. Int J Sports Med. 1990;11(1):26–32. 10.1055/s-2007-1024757 2318561

[32] Grant S, McMillan K, Newell J, Wood L, Keatley S, Simpson D, et al Reproducibility of the blood lactate threshold, 4 mmol.l(-1) marker, heart rate and ratings of perceived exertion during incremental treadmill exercise in humans. Eur J Appl Physiol. 2002;87(2):159–66. 10.1007/s00421-002-0608-2 12070627

[33] Pfitzinger P, Freedson PS. The reliability of lactate measurements during exercise. Int J Sports Med. 1998;19(5):349–57. 10.1055/s-2007-971929 9721059

[34] Pallares JG, Moran-Navarro R, Ortega JF, Fernandez-Elias VE, Mora-Rodriguez R. Validity and Reliability of Ventilatory and Blood Lactate Thresholds in Well-Trained Cyclists. PLoS One. 2016;11(9):e0163389 10.1371/journal.pone.0163389 27657502 PMC5033582

[35] Borszcz FK, Tramontin AF, de Souza KM, Carminatti LJ, Costa VP. Physiological Correlations With Short, Medium, and Long Cycling Time-Trial Performance. Research quarterly for exercise and sport. 2018;89(1):120–5. 10.1080/02701367.2017.1411578 29334005

[36] Nichols JF, Phares SL, Buono MJ. Relationship between blood lactate response to exercise and endurance performance in competitive female master cyclists. Int J Sports Med. 1997;18(6):458–63. 10.1055/s-2007-972664 9351693

[37] Bentley DJ, McNaughton LR, Thompson D, Vleck VE, Batterham AM. Peak power output, the lactate threshold, and time trial performance in cyclists. Med Sci Sports Exerc. 2001;33(12):2077–81. 11740302 10.1097/00005768-200112000-00016

[38] McNaughton LR, Roberts S, Bentley DJ. The relationship among peak power output, lactate threshold, and short-distance cycling performance: effects of incremental exercise test design. J Strength Cond Res. 2006;20(1):157–61. 10.1519/R-15914.1 16506862

