# Supplementary material for: Repeatability and predictive value of lactate threshold concepts in endurance sports
Source: PLoS One. 2018 Nov 14;13(11):e0206846. doi: 10.1371/journal.pone.0206846 (PMC6235347; doi:10.1371/journal.pone.0206846)
Supplement: S1 Table — (DOCX) [file pone.0206846.s001.docx]

# Supporting information

Supplemental Table 1. Lactate threshold concept categories

| **Category** | **Method** |
| --- | --- |
| *Fixed bLa value* | [LT-4mmol] Power at 4 mmol/L (1-5) |
|  | Power at 2, 2.5, 4 mmol/L (6, 7) |
|  | Power at 2.5, 3, 4 mmol/L (8) |
|  | Power at 2.5 mmol/L (9) |
|  | Power at 2 mmol/L (3) |
| *Visually determined rise from baseline* | [LT1] First rise of bLa above baseline (3) |
|  | Just before onset of lactate accumulation (10) |
|  | First breakpoint in lactate accumulation (11) |
|  | Systematic increase above baseline (12) |
|  | Marked increase above resting baseline (13) |
|  | Begin of increase above resting level (3) |
|  | Increase above resting level (14) |
|  | Velocity before observation of sudden-sustained increase in bLa (15) |
|  | Log(bLa) vs log(VO_2_), regression points visually determined (16) |
| *Predetermined increase* | First 0.2 mmol/L increase above baseline (6, 7) |
|  | First 0.5 mmol/L increase above baseline (17) |
|  | [LT2] First 1 mmol/L increase above baseline (3, 14) |
|  | First 1 mmol/L increase above baseline at 40-60% VO_2_max (18) |
|  | First significant rise of 2 mmol/L (4) |
|  | Second lactate increase of at least 0.5mmol/L with second increase ≥ first increase (19) |
|  | [LT4] Preceding a bLa increase by 1mmol/L or more (20, 21) |
|  | Rise in delta lactate (22) |
| *Intersection of tangents and other derivatives* | Intersection between tangent for minimum lactate equivalent and Linear function of final 90s (23) |
|  | Perpendicular bisection of BLC from intersection of tangent for upper and lower bLa values (24) |
|  | [Dmax] Dmax method (25) |
|  | [Dmax-mod] Dmax modified method (20) |
| *Minimum lactate equivalent* | Lowest value of bLa/running speed (26) |
|  | [LT5] Lowest value of bLa/VO2/kg (27) |
|  | [LT3] 1.5 mmol/L above minimum lactate equivalent (lowest value for bLa/W) (27, 28) |
| *Not selected* | |
| *Using the recovery bLa curve* | Tangent to bLa curve from recovery curve where bLa is equal to the value at end of GXT (29) |
|  | Lowest bLa in GXT after high exercise (30) |
| *Tangent to bLa curve* | Tangent to bLa curve at 51 degrees (31) |
|  | Tangent to bLa curve at 45 degrees (32) |

In the column Method, selected concepts are indicated in [brackets].

References

1. Sjodin B, Jacobs I. Onset of blood lactate accumulation and marathon running performance. Int J Sports Med. 1981;2(1):23-6.

2. Mader A LH, Heck H,. Zur Beurteilung der sportartspezifischen ausdauerleistungsfa¨ higkeit im labor. Sportarzt Sportmed. 1979(27):80-8, 109-12.

3. Yoshida T, Chida M, Ichioka M, Suda Y. Blood lactate parameters related to aerobic capacity and endurance performance. Eur J Appl Physiol Occup Physiol. 1987;56(1):7-11.

4. Kindermann W, Simon G, Keul J. The significance of the aerobic-anaerobic transition for the determination of work load intensities during endurance training. Eur J Appl Physiol Occup Physiol. 1979;42(1):25-34.

5. Yoshida T, Udo M, Iwai K, Chida M, Ichioka M, Nakadomo F, et al. Significance of the contribution of aerobic and anaerobic components to several distance running performances in female athletes. Eur J Appl Physiol Occup Physiol. 1990;60(4):249-53.

6. Weltman J, Seip R, Levine S, Snead D, Rogol A, Weltman A. Prediction of lactate threshold and fixed blood lactate concentrations from 3200-m time trial running performance in untrained females. Int J Sports Med. 1989;10(3):207-11.

7. Weltman A, Snead D, Seip R, Schurrer R, Levine S, Rutt R, et al. Prediction of lactate threshold and fixed blood lactate concentrations from 3200-m running performance in male runners. Int J Sports Med. 1987;8(6):401-6.

8. Fohrenbach R, Mader A, Hollmann W. Determination of endurance capacity and prediction of exercise intensities for training and competition in marathon runners. Int J Sports Med. 1987;8(1):11-8.

9. Hurley BF, Hagberg JM, Allen WK, Seals DR, Young JC, Cuddihee RW, et al. Effect of training on blood lactate levels during submaximal exercise. J Appl Physiol Respir Environ Exerc Physiol. 1984;56(5):1260-4.

10. Ivy JL, Withers RT, Van Handel PJ, Elger DH, Costill DL. Muscle respiratory capacity and fiber type as determinants of the lactate threshold. J Appl Physiol Respir Environ Exerc Physiol. 1980;48(3):523-7.

11. Tanaka H. Predicting running velocity at blood lactate threshold from running performance tests in adolescent boys. Eur J Appl Physiol Occup Physiol. 1986;55(4):344-8.

12. Tanaka K, Matsuura Y. Marathon performance, anaerobic threshold, and onset of blood lactate accumulation. J Appl Physiol Respir Environ Exerc Physiol. 1984;57(3):640-3.

13. Tanaka K, Matsuura Y, Matsuzaka A, Hirakoba K, Kumagai S, Sun SO, et al. A longitudinal assessment of anaerobic threshold and distance-running performance. Med Sci Sports Exerc. 1984;16(3):278-82.

14. Coyle EF, Martin WH, Ehsani AA, Hagberg JM, Bloomfield SA, Sinacore DR, et al. Blood lactate threshold in some well-trained ischemic heart disease patients. J Appl Physiol Respir Environ Exerc Physiol. 1983;54(1):18-23.

15. Smith CG, Jones AM. The relationship between critical velocity, maximal lactate steady-state velocity and lactate turnpoint velocity in runners. Eur J Appl Physiol. 2001;85(1-2):19-26.

16. Beaver WL, Wasserman K, Whipp BJ. Improved detection of lactate threshold during exercise using a log-log transformation. J Appl Physiol (1985). 1985;59(6):1936-40.

17. Hughson RL, Green HJ. Blood acid-base and lactate relationships studied by ramp work tests. Med Sci Sports Exerc. 1982;14(4):297-302.

18. Hagberg JM, Coyle EF. Physiological determinants of endurance performance as studied in competitive racewalkers. Med Sci Sports Exerc. 1983;15(4):287-9.

19. Baldari C, Guidetti L. A simple method for individual anaerobic threshold as predictor of max lactate steady state. Med Sci Sports Exerc. 2000;32(10):1798-802.

20. Bishop D, Jenkins DG, Mackinnon LT. The relationship between plasma lactate parameters, Wpeak and 1-h cycling performance in women. Med Sci Sports Exerc. 1998;30(8):1270-5.

21. Amann M, Subudhi AW, Foster C. Predictive validity of ventilatory and lactate thresholds for cycling time trial performance. Scand J Med Sci Sports. 2006;16(1):27-34.

22. Farrell PA, Wilmore JH, Coyle EF, Billing JE, Costill DL. Plasma lactate accumulation and distance running performance. Med Sci Sports. 1979;11(4):338-44.

23. Berg A SJ, Keul J,. Zur Beurteilung der Leistungsfa¨ higkeit und Belastbarkeit von Patienten mit coronarer Herzkrankheit. Dtsch Z Sportmed. 1980(31):199-205.

24. Bunc V HJ, Novack J,. Determination of the individual anaerobic threshold. Acta Univ Carol, Gymnica. 1985(27):73-81.

25. Cheng B, Kuipers H, Snyder AC, Keizer HA, Jeukendrup A, Hesselink M. A new approach for the determination of ventilatory and lactate thresholds. Int J Sports Med. 1992;13(7):518-22.

26. Roecker K, Schotte O, Niess AM, Horstmann T, Dickhuth HH. Predicting competition performance in long-distance running by means of a treadmill test. Med Sci Sports Exerc. 1998;30(10):1552-7.

27. Dickhuth H.-H.; Huonker M. MT, Drexler H., Berg A., Keul J. Individual anaerobic threshold for evaluation of competitive athletes and patients with left ventricular dysfunctions. Advances in ergometry. 1991.

28. Dickhuth H-H, Yin L, Niess A, Rocker K, Mayer F, Heitkamp HC, et al. Ventilatory, lactate-derived and catecholamine thresholds during incremental treadmill running: relationship and reproducibility. Int J Sports Med. 1999;20(2):122-7.

29. Stegmann H, Kindermann W, Schnabel A. Lactate kinetics and individual anaerobic threshold. Int J Sports Med. 1981;2(3):160-5.

30. Tegtbur U, Busse MW, Braumann KM. Estimation of an individual equilibrium between lactate production and catabolism during exercise. Med Sci Sports Exerc. 1993;25(5):620-7.

31. Keul J SG, Berg A, et al. Bestimmung der individuellen anaeroben Schwelle zur Leistungsbewertung und Trainingsgestaltung. Dtsch Z Sportmed. 1979(30):212-8.

32. Simon G BA, Dickhuth H-H,. Bestimmung der anaeroben Schwelle in Abha¨ ngigkeit von Alter und von der Leistungsfa¨ higkeit. Dtsch Z Sportmed. 1981(32):7-14.
