# Supplementary material for: Repeatability and predictive value of lactate threshold concepts in endurance sports
Source: PLoS One. 2018 Nov 14;13(11):e0206846. doi: 10.1371/journal.pone.0206846 (PMC6235347; doi:10.1371/journal.pone.0206846)
Supplement: S1 Protocol — (PDF) [file pone.0206846.s002.pdf]

CHDR
Centre for Human Drug Research

CLINICAL STUDY PROTOCOL

A randomized, double-blind, placebo-controlled study to investigate the effects of recombinant human erythropoietin (NeoRecormon) on cycling performance and the occurrence of adverse events in well-trained cyclists.

Short Title:	Recombinant human erythropoietin effects on cycling performance.
Version:	6
Date:	17-May-2016
CHDR number:	CHDR1514
Toetsing Online number:	NL54516.056.15
EudraCT number:	2015-003269-27

CONTACT DETAILS

**Trial Site**<br>**(visit & delivery address)**	Centre for Human Drug Research<br>Zernikedreef 8<br>2333 CL Leiden<br>The Netherlands<br>Telephone: + 31 71 5246 400<br>Fax: + 31 71 5246 499<br>Emergency: + 31 71 5246 444
Principal Investigator	A.F. (Adam) Cohen, MD, PhD<br>Telephone: + 31 (0)71 5246404<br>e-mail: ac@chdr.nl
Co-Investigator	J. (Koos) Burggraaf, MD, PhD<br>Telephone: + 31 (0)71 5246418<br>e-mail: kb@chdr.nl
Co-Investigator	Dr. J.I. (Joris) Rotmans<br>Telephone: + 31 71 5262148<br>e-mail: J.I.Rotmans@lumc.nl
Co-Investigator	Dr. J.M.A. (Hans) Daniels<br>Telephone: +31 (0)20 4444782<br>e-mail: j.daniels@vumc.nl
Co-Investigator	J. A. A. C. (Jules) Heuberger, MSc<br>Telephone: +31 (0)71 5246471<br>e-mail: JHeuberger@chdr.nl
Co-Investigator	Mr. H. (Herman) Ram<br>Telephone: 010 - 2010154<br>e-mail: H.Ram@dopingautoriteit.nl
Co-Investigator	Dr. O. (Olivier) de Hon<br>Telephone: 010-2010155<br>e-mail: O.Dehon@dopingautoriteit.nl
Co-Investigator	T. (Thijs) Zonneveld<br>e-mail: info@thijszonneveld.com
Director of Clinical Operations	J. M. (Ria) Kroon, BSc<br>Telephone: + 31 (0)71 5246498<br>e-mail: rk@chdr.nl
Statistician	M. L. (Marieke) de Kam, MSc<br>Telephone: + 31 (0)71 5246458<br>e-mail: MdeKam@chdr.nl

**INDEPENDENT PHYSICIAN**	Prof. Dr G.J. Blauw, MD, PhD<br>Department of Gerontology and Geriatrics LUMC<br>Postbus 9600<br>2300 RC Leiden<br>Telephone: + 31 71 5266 640
**LABORATORY - CHEMISTRY**	C.M. Cobbaert, PhD<br>CKCL LUMC, E2-P<br>Albinusdreef 2<br>2333 ZA Leiden<br>The Netherlands
Contact person	J. Verhagen
**LABORATORY - HAEMATOLOGY**	W.A.F. Marijt, MD, PhD<br>CKHL LUMC, E1-Q<br>Albinusdreef 2<br>2333 ZA Leiden<br>The Netherlands
Contact person	F. Reymer
**LABORATORY – MICROBIOLOGY**	A.C.M. Kroes, MD, PhD<br>KML LUMC, E4-P<br>Albinusdreef 2<br>2333 ZA Leiden<br>The Netherlands
Contact person	A.C.M. Kroes
**LABORATORY – DOPING DETECTION**[	DoCoLab – Ugent<br>Technologiepark 30<br>B-9052, Zwijnaarde<br>Belgium
Contact person	Prof. Dr. ir. Peter Van Eenoo<br>Telephone: + 32-9-3313291<br>e-mail: Peter.VanEenoo@Ugent.be
**LABORATORY – RNA EXPRESSION**	Leiden Genome Technology Center (LGTC)<br>Human and Clinical Genetics<br>Leiden University Medical Center, Postzone S4-P<br>Postbus 9600<br>2300 RC Leiden<br>Nederland

Contact person	Henk Buermans<br>Tel: +31-71-526 9500/9522<br>Fax: +31-71-526 8285<br>E-mail: info@LGTC.nl
**PHARMACY**<br>**(visit & delivery address)**	Apotheek LUMC, L0-P30<br>Albinusdreef 2<br>2333 ZA Leiden<br>The Netherlands<br>Telephone: + 31 71 5269111<br>Fax + 31 71 5262611
Pharmacist / Trial Manager	Irene Teepe-Twiss, PhD / Linda van der Hulst

SIGNATURE PAGE - PRINCIPAL INVESTIGATOR

Study Title

A randomized, double-blind, placebo-controlled study to investigate the effects of recombinant human erythropoietin (NeoRecormon) on cycling performance and the occurrence of adverse events in well-trained cyclists.

I acknowledge accountability for this protocol in accordance with CHDR's current procedures.

A.F. (Adam) Cohen, MD, PhD<br>Principal Investigator	[signature]	20/5/2016
	Signature	Date (dd Mmm yyyy)

SIGNATURE PAGE - TRIAL SITE STAFF
Centre for Human Drug Research

Study Title

A randomized, double-blind, placebo-controlled study to investigate the effects of recombinant human erythropoietin (NeoRecormon) on cycling performance and the occurrence of adverse events in well-trained cyclists.

I acknowledge responsibility for this protocol in accordance with CHDR's current procedures.

J.A.A.C (Jules) Heuberger, MSc<br>Co-Investigator	[signature]<br>Signature	20 May 2016<br>Date (dd Mmm yyyy)
J. M. (Ria) Kroon, BSc<br>Director Clinical Operations	pp [signature]<br>Signature	20/5/2016<br>Date (dd Mmm yyyy)
M. L. (Marieke) de Kam, MSc<br>Statistician	[signature] RI<br>Signature<br>In absence of.	20 May 2016<br>Date (dd Mmm yyyy)

TABLE OF CONTENTS

CONTACT DETAILS **2**

SIGNATURE PAGE - PRINCIPAL INVESTIGATOR **5**

SIGNATURE PAGE - TRIAL SITE STAFF **6**

PROTOCOL SYNOPSIS – CHDR1514 ..... **13**

1 BACKGROUND AND RATIONALE **23**

1.1 Context 23

1.1.1 Need for research on doping 23

1.1.2 Erythropoietin substances in patients and athletes 23

1.1.3 Examination of the evidence for the ergogenic properties of rHuEPO in cyclists 23

1.1.4 Study objectives 24

1.2 Non-clinical information 24

1.2.1 Non-clinical pharmacology 24

1.2.2 Non-clinical pharmacokinetics and metabolism 24

1.2.3 Non-clinical toxicology and safety pharmacology 24

1.3 Clinical information 24

1.3.1 Clinical pharmacology 24

1.3.2 Clinical pharmacokinetics and metabolism 24

1.3.3 Clinical toxicology and safety pharmacology 24

1.4 Study rationale 24

1.4.1 Benefit and risk assessment 24

1.4.2 Medical and regulatory background 25

1.4.3 Study population 25

1.4.4 Study design 25

1.4.5 Investigational drug and placebo 26

1.4.6 Dosing, safety margin calculations, stopping criteria 26

1.4.7 Treatment duration 27

1.4.8 Endpoints 27

1.4.9 Statistical hypotheses and sample size 28

2 STUDY OBJECTIVES **30**

2.1 Primary objective 30

2.2 Secondary objectives 30

2.3 Exploratory objectives 30

3 STUDY DESIGN **31**

3.1 Overall study design and plan 31

3.1.1 Screening 31

3.1.2 Treatment and exercise tests 32

3.1.3 Training period 32

3.1.4 Competition 32
3.1.5 Follow-up 32
3.1.6 Urine doping control 32
4 STUDY POPULATION 33
4.1 Subject population 33
4.2 Inclusion criteria 33
4.3 Exclusion criteria 33
4.4 Concomitant medications 34
4.4.1 Mandatory concomitant supplementation 34
4.4.2 Allowed concomitant medications 34
4.4.3 Prohibited concomitant medications 34
4.5 Lifestyle restrictions 34
4.6 Study drug discontinuation and withdrawal 35
4.6.1 Study drug interruption or discontinuation 35
4.6.2 Subject withdrawal 35
4.6.3 Replacement policy 35
5 INVESTIGATIONAL MEDICINAL PRODUCT 36
5.1 Investigational drug and matching placebo 36
5.2 Comparative drug 36
5.3 Study drug dosing scheme 36
5.4 Study drug packaging and labelling 36
5.5 Drug accountability 36
5.6 Treatment assignment and blinding 36
5.6.1 Treatment assignment 36
5.6.2 Blinding 37
6 STUDY ENDPOINTS 38
6.1 Efficacy endpoints 38
6.2 Safety endpoints 39
6.3 Exploratory endpoints 40
7 STUDY ASSESSMENTS 41
7.1 Exercise-specific screening assessments 41
7.1.1 Exercise test 41
7.1.2 Questionnaire 41
7.2 Safety and tolerability assessments 41
7.2.1 Specific safety assessments 41
7.2.2 Vital signs 41
7.2.3 Weight and height 41
7.2.4 Physical examination 41

7.2.5 Electrocardiography ... 41
7.2.6 Laboratory assessments ... 41
7.3 Efficacy assessments ... 43
7.3.1 Exercise tests ... 43
7.3.2 Competition ... 43
7.3.3 Athlete biological passport ... 43
7.3.4 Blood flow ... 43
7.4 NeoRecormon detection assessments ... 43
7.4.1 Urine doping screen ... 43
7.5 RNA expression levels ... 43
7.6 Sequence of assessments and time windows ... 44
7.7 Total blood volume ... 45
8 SAFETY REPORTING ... 46
8.1 Definitions of adverse events ... 46
8.1.1 Intensity of adverse events ... 46
8.1.2 Relationship to study drug ... 46
8.1.3 Chronicity of adverse events ... 46
8.1.4 Action ... 46
8.1.5 Serious adverse events ... 46
8.1.6 Suspected unexpected serious adverse reactions ... 47
8.1.7 Reporting of serious adverse events ... 47
8.1.8 Follow-up of adverse events ... 47
8.2 Section 10 WMO event ... 47
8.3 Annual safety report or development safety update report ... 47
9 STATISTICAL METHODOLOGY AND ANALYSES ... 49
9.1 Statistical analysis plan ... 49
9.2 Protocol violations/deviations ... 49
9.3 Power calculation ... 49
9.4 Missing, unused and spurious data ... 49
9.5 Analysis sets ... 49
9.5.1 Safety set ... 49
9.5.2 Efficacy analysis set ... 49
9.6 Subject disposition ... 49
9.7 Baseline parameters and concomitant medications ... 50
9.7.1 Demographics and baseline variables ... 50
9.7.2 Medical history ... 50
9.7.3 Concomitant Medications ... 50
9.7.4 Treatment compliance/exposure ... 50

9.8 Safety and tolerability endpoints ... 50
9.8.1 Adverse events ... 50
9.8.2 Vital signs ... 50
9.8.3 ECG ... 51
9.8.4 Clinical laboratory tests ... 51
9.9 Efficacy endpoints ... 51
9.9.1 Efficacy ... 51
9.9.2 Inferential methods ... 51
9.10 Exploratory analyses and deviations ... 52
9.11 Interim analyses ... 52
10 GOOD CLINICAL PRACTICE, ETHICS AND ADMINISTRATIVE PROCEDURES ... 53
10.1 Good clinical practice ... 53
10.1.1 Ethics and good clinical practice ... 53
10.1.2 Ethics committee / institutional review board ... 53
10.1.3 Informed consent ... 53
10.1.4 Insurance ... 53
10.2 Study funding ... 53
10.3 Data handling and record keeping ... 53
10.3.1 Data collection ... 53
10.3.2 Database management and quality control ... 54
10.4 Access to source data and documents ... 54
10.5 Quality control and quality assurance ... 54
10.5.1 Monitoring ... 54
10.6 Protocol amendments ... 54
10.6.1 Non-substantial amendment ... 54
10.6.2 Substantial amendment ... 54
10.7 End of study report ... 55
10.8 Public disclosure and publication policy ... 55
11 STRUCTURED RISK ANALYSIS ... 56
12 REFERENCES ... 57
13 APPENDIX 1 – SUMMARY OF PRODUCT CHARACTERISTICS – ENGLISH VERSION ... 59
14 APPENDIX 2 – SUMMARY OF PRODUCT CHARACTERISTICS – DUTCH VERSION ... 60
15 APPENDIX 3 – EUROPEAN PUBLIC ASSESSMENT REPORT ... 61
16 APPENDIX 4 – SCREENING: QUESTIONNAIRE SPORT ACTIVITIES ... 62
17 APPENDIX 5 – EXERCISE TEST ... 64
18 APPENDIX 6 – COMPETITION ... 65
19 APPENDIX 7 – URINE DOPING CONTROL PROCEDURE ... 67

LIST OF ABBREVIATIONS

AE	Adverse Event
ALT	Alanine aminotransferase/Serum glutamic pyruvic transaminase (SGPT)
ANCOVA	Analysis of Covariance
ANOVA	Analysis of Variance
ATC	Anatomic Therapeutic Chemical
ABP	Athletes Biological Passport
BMI	Body Mass Index
BP	Blood Pressure
bpm	Beats per minute
CCMO	Central Committee on Research Involving Human Subjects; in Dutch: Centrale Commissie Mensgebonden Onderzoek
CHDR	Centre for Human Drug Research
CIRC	Cycling Independent Reform Commission
CK	Creatine Kinase
CRF	Case Report Form
EC	Ethics Committee (also Medical Research Ethics Committee (MREC); in Dutch: Medisch Ethische Toetsing Commissie (METC).
ECG	Electrocardiogram
EDTA	Ethylene diamine tetra-acetic acid
FDA	Food and Drug Administration
GCP	Good Clinical Practice
Hb	Haemoglobin
Ht	Haematocrit
ICH	International Conference on Harmonization
IRB	Institutional Review Board
LDH	Lactate dehydrogenase
MedDRA	Medical Dictionary for Regulatory Activities
rHuEPO	Recombinant Human Erythropoietin
SAE	Serious Adverse Event
SAP	Statistical Analysis Plan
SD	Standard Deviation
SEM	Standard Error of the Mean
SOC	System Organ Class
SOP	Standard Operating Procedure
SST	Serum Separator Tube
SmPC	Summary of Product Characteristics
SUSAR	Suspected Unexpected Serious Adverse Reaction
TTE	Time To Exhaustion
UCI	Union Cycliste Internationale
WADA	World Anti-Doping Agency
WHO	World Health Organization
WMO	Medical Research Involving Human Subjects Act; in Dutch: Wet Medisch-wetenschappelijk Onderzoek met Mensen.

PROTOCOL SYNOPSIS – CHDR1514

1. Title

A randomized, double-blind, placebo-controlled study to investigate the effects of recombinant human erythropoietin (NeoRecormon) on cycling performance and the occurrence of adverse events in well-trained cyclists.

2. Short Title

Recombinant human erythropoietin effects on cycling performance.

3. Background & Rationale

3.1 Need for research on doping

A recent report of the Union Cycliste Internationale gives an in-depth analysis of doping throughout cycling's history, from 1890 to the present day. The report's final conclusion is that cycling has had, and continues to have, a serious doping problem.[1]

Although it could be argued that administering substances that improve performance is forbidden and nothing more needs to be known about it, these substances are apparently being used and therefore research to investigate the effects and safety of doping substances in this population is necessary.

There are a number of reasons for this. First, it is often unknown if a forbidden substance really improves performance. If this is not the case the need for administration is strongly diminished. Additionally the adverse effects of such substances are often insufficiently known and athletes may be exposed to risks without being adequately informed about them.

3.2 Erythropoietin substances in patients and athletes

Recombinant Human Erythropoietin (rHuEPO) is used to treat patients with anemia resulting from chronic kidney disease or chemotherapy.[2] The correction of the anemia results in an increase in exercise capacity in these patients.[2] The treatment immediately attracted the attention of athletes because they assumed that rHuEPO would also improve their exercise performance. Due to this presumption, the use of rHuEPO in athletes became very common. In 1990, the use of rHuEPO was placed on the list of prohibited substances published by the World Anti-Doping Agency (WADA).[3] At the time of the first ban there was no published evidence that rHuEPO would actually improve sports performance.

3.3 Examination of the evidence for the ergogenic properties of rHuEPO in cyclists

The evidence for the effect of rHuEPO in well trained athletes is in fact sparse until today. A qualitative systematic review of the available literature was performed in 2012 to examine the evidence for the performance enhancing properties of rHuEPO in cyclists.[4]

The review demonstrated that the characteristics of the study populations differed from the population suspected of rHuEPO abuse. Studies did not use well-trained cyclists, still less elite or world-class cyclists.[5] Most studies used a small number of untrained subjects and the quality of the research was often questionable. In these studies, the main studied effect was the maximal oxygen carrying capacity of blood ($V_{O2,\ max}$) which only has a remote connection to performance in endurance sports, especially in well-trained athletes. This is in line with the knowledge that multiple factors affect performance, in which oxygen carrying capacity of the blood becomes less relevant when other factors become rate-limiting. Endurance performance may be better correlated with submaximal exercise factors. In addition, there is virtually no research on the potential adverse effects of this form of doping.

To conclude, the results of this literature search showed that there is no scientific basis from which to conclude that rHuEPO has performance-enhancing properties in well-trained cyclists and that knowledge about potential side effects is lacking in full.

3.4 Aim

In the current study the effect of NeoRecormon, a rHuEPO, will be investigated in a population with

cycling performance abilities as close as possible to those of professional cyclists and in conditions closely resembling racing conditions and the required performance duration.

4. Objective(s)

4.1 Primary Objective

- To explore the effects of NeoRecormon on well-trained cyclists and their cycling performance by maximal and sub-maximal exercise parameters measured during exercise tests.

4.2 Secondary Objectives

- To explore the effects of NeoRecormon on well-trained cyclists and their cycling performance by the performance and outcome of a race.
- To evaluate the safety of NeoRecormon in well-trained cyclists.
- To evaluate the performance of doping detection methods for NeoRecormon use in well-trained cyclists.

4.3 Exploratory objectives

- To explore how a standardized submaximal exercise affects gene expression patterns in well-trained individuals.
- To explore the difference in RNA-profiles between individuals treated with rHuEPO and placebo
- To identify potential transcripts that can be used as biomarkers for rHuEPO use
- To explore correlations between changes in whole blood gene expression patterns observed before and after a submaximal exercise test in individuals and their performance

5. Design

Randomized, double-blind, placebo-controlled study to investigate the effects and safety of NeoRecormon in well-trained cyclists.

6. Principal Investigator & Trial Site

Principal Investigator:	Prof. dr. A.F. (Adam) Cohen, MD
Co-Investigators:	Prof. dr. J. (Koos) Burggraaf, MD<br>Dr. J.I. (Joris) Rotmans<br>Dr. J.M.A. (Hans) Daniels<br>J.A.A.C. (Jules) Heuberger, MSc<br>Mr. H. (Herman) Ram<br>Dr. O. (Olivier) de Hon<br>T. (Thijs) Zonneveld
Trial Site:	Centre for Human Drug Research, Zernikedreef 8, 2333 CL Leiden, The Netherlands

7. Subjects / Groups

A total of 48 subjects are planned to be enrolled in this study. Eligible subjects will be randomized to the NeoRecormon or Placebo treatment groups on a 1:1 basis.

8. Sample Size Justification

8.1 Power analysis based on $V_{O2,max}$

Based on the increase in $V_{O2,max}$ after administration of NeoRecormon in a previous study with moderately trained subjects an exploratory power analysis has been performed.[6] A sample size of 6 in each group will have a power of 80% to detect a difference in means of 3.8 ml/min/kg, assuming that the common standard deviation is 1.95, using a two-tailed t-test with a 0.05 two sided significance level.

A review of the available literature showed that, after initial years of training, well-trained athletes maintain a plateau in their $V_{O2,max}$, but continue to improve their performance.[4] This indicates that the difference between effects on $V_{O2,max}$ between the NeoRecormon and placebo group in well-

trained cyclists might be smaller. The smaller the size of the difference, the larger the sample size must be to detect a significant difference. To detect a difference of 1.5ml/min/kg with a power of 80% a sample size of 22 is needed, assuming that the common standard deviation is 1.95, using a two-tailed t-test with a 0.05 two sided significance level. When taking into account a ±10% attrition rate, 24 subjects are needed in both the NeoRecormon and placebo group.

8.2 Power analysis based on P_{max}/kg

The power calculation in section 8.1 is based on an endpoint that only has a remote connection to performance in well-trained cyclists. A better endpoint would be power output per kilogram (P/kg) at a submaximal level, such as 80% of $V_{O2,max}$. Unfortunately no studies have been performed using this endpoint, so the effect of rHuEPO on P/kg at 80% $V_{O2,max}$ is still unknown. The mean P/kg at 80% $V_{O2,max}$ of 11 male professional cyclists however, is 5.2 W/kg with a standard deviation of 0.199.[7] Using a sample size of 22 (including 10% attrition rate) and a two-tailed t-test with a 0.05 two sided significance level a difference of 0.172 W/kg can be detected with a power of 80%. This difference would mean that a professional cyclist weighing 75 kg would go from an average of 390 W at 80% $V_{O2,max}$ to 402.9 W. On a racing bike weighing 9 kg sitting in racing position at 25 degrees Celsius, this would produce a speed of 43.80 km/h and 44.32 km/h respectively (calculated from http://bikecalculator.com). In a flat terrain of 40 km this would result in a finish time of 54 min 48 sec and 54 min 09 sec, a difference of 39 seconds, which is very relevant in a race like to Tour the France.

9. Inclusion criteria

- Well-trained (as determined by cycling history and maximal power output >4 W/kg) male subjects, 18 to 50 years old (inclusive);
- Subjects must be healthy / medically stable on the basis of clinical laboratory tests, medical history, vital signs, and 12-lead ECG performed at screening, including exercise ECG.
- Each subject must sign an informed consent form prior to the study. This means the subject understands the purpose of and procedures required for the study.

10. Exclusion criteria

- Any clinically significant abnormality, as determined by medical history taking and physical examinations, obtained during the screening visit that in the opinion of the investigator would interfere with the study objectives or compromise subject safety.
- Unacceptable known concomitant diagnoses or diseases at baseline, e.g., known cardiovascular, pulmonary, muscle, metabolic or haematological disease, renal or liver dysfunction, ECG or laboratory abnormalities, etc.
- Unacceptable concomitant medications at baseline, e.g., drugs known or likely to interact with the study drugs or study assessments.
- Unacceptable potential cycling performance enhancing medications at baseline, e.g. Erythropoiesis-stimulating agents, Anabolic Androgenic Steroids, Growth Hormone, Insulin, IGF-I and Beta-Adrenergic Agents or methods, e.g. altitude tents.
- Blood transfusion in the past three months.
- Loss or donation of blood over 500 mL within three months.
- Participation in a clinical trial within 90 days of screening or more than 4 times in the previous year.
- Known hypersensitivity to the treatment or drugs of the same class, or any of their excipients.
- Any known factor, condition, or disease that might interfere with treatment compliance, study conduct or interpretation of the results such as drug or alcohol dependence or psychiatric disease.
- Positive urine drug test at screening.

- Positive alcohol breath test at screening.
- Haemoglobin (Hb) concentration > 9.8 mmol/l at screening.
- Hb concentration < 8 mmol/l at screening.
- Haematocrit (Ht) ≥ 48% at screening.
- Being subject to WADA's anti-doping rules, meaning being a member of an official cycling union or other sports union for competition (such as the KNWU) or participating in official competition during the study.
- Positive results from serology at screening (except for vaccinated subjects or subjects with past but resolved hepatitis)
- Previous history of fainting, collapse, syncope, orthostatic hypotension, or vasovagal reactions.
- Any circumstances or conditions, which, in the opinion of the investigator, may affect full participation in the study or compliance with the protocol.

11. Concomitant medications

The clinical results obtained so far do not indicate any interaction of NeoRecormon with other medicinal products.

Mandatory supplementation:

- 50mg vitamin C (ascorbic acid) per day
- 200 mg iron (ferrofumerate) per day

Allowed:

- Paracetamol
- Other medications that are discussed, approved and clearly documented by the investigator.

Prohibited:

- All substances (except NeoRecormon during treatment period) that are on the doping list and enhance cycling performance are prohibited within 6 months prior to study drug administration and during the course of the study (e.g. Other Erythropoiesis-stimulating agents, Anabolic Androgenic Steroids, Growth Hormone, Insulin, IGF-I and Beta-Adrenergic Agents).

12. Study periods

The total study period will be 17 weeks.

Study periods	Occasion	Weeks
Screening + training	1x	Within 6 weeks prior to treatment period
Ramp exercise test*:	1x	Within 2 weeks before the start of the treatment period, but after the screening
Time To Exhaustion exercise test*:	1x	
Treatment	Once a week	During 8 weeks
Ramp exercise test	Every two weeks	
Training	Whole period	
TTE exercise test:	1x	During the 7th treatment week
Competition	1x	During the 9th week
Follow-up	1x	30 Days after last visit

*Exercise tests will be performed within 2 weeks prior to the treatment period for baseline measurements.

13. Investigational drug

rHuEPO: NeoRecormon (Active substance: Epoëtine beta)

Dosage: See Figure 1 for a detailed description of the dosage adjustment schedule.

Administration: Subcutaneously

Dosage Rationale: NeoRecormon 2000, 5000 or ≥6000 but ≤10.000 IU/week, to be able to reach the target range and adjust the dosage as soon as possible if it seems necessary from Hb or Ht results

14. Placebo

Placebo: subcutanous injection of saline, (0.90% w/v NaCl)

The investigational drug and its matching placebo are indistinguishable and will be packaged in the same way. Blinding will be accomplished by using the same syringes or by covering the syringes with aluminum foil.

15. Efficacy endpoints

Efficacy will be assessed at the time points indicated in the Visit and Assessment Schedule (see Table 1) in four ways:

- Exercise tests will measure maximal and submaximal exercise parameters (e.g. $V_{O2,max}$, P_{max}, V_E, V_t, V_{O2}, V_{CO2}, see

- **Table 2.**)
- Subjects will participate in a competition designed in such a way that it closely resembles real racing conditions. Before and during the race, physiological parameters will be measured (e.g. power, heart rate, blood pressure).
- Blood will be collected at predetermined stages at each clinical visit before administration of NeoRecormon/placebo and before and during the exercise tests and competition. This blood will be used for the following Clinical Laboratory Assessments:
  - Haematology (e.g. markers for the Athlete's Biological Passport)
  - Chemistry (e.g. creatinine phosphokinase and c-reactive protein levels)
  - Coagulation (e.g. D-Dimer, F1+2, e-Selectin, p-Selectin)
- Laser Speckle contrast imaging for blood flow measurements.

16. Safety endpoints

Safety will be assessed by:

1. Physical examination
2. Monitoring vital signs
   - o Pulse Rate (bpm)
   - o Systolic blood pressure (mmHg)
   - o Diastolic blood pressure (mmHg)
   - o Temperature measurements (ºC)
3. Electrocardiogram (ECG)
   - o Heart Rate (HR) (bpm), PR, QRS, QT, QTcB
4. Clinical Laboratory Assessments
   - o Haematology
     Ht must be <52%. If Ht level is ≥52%, therapy should be interrupted until the Ht percentage begins to fall.
     Hb must be below a certain level (see Figure 1). If Hb exceeds that level, therapy should be interrupted until the Hb concentration falls back into the range.
   - o Chemistry
   - o Urinalysis
   - o Coagulation
5. Collection of treatment-emergent (serious) adverse events ((S)AEs)

1-4 will be assessed during screening, 2-5 will be assessed at each clinical visit and before the competition. 2 and 3 will be assessed before and during the exercise tests.

(Serious) Adverse Events ((S)AEs) will be collected throughout the study.

17. Blinding

This study will be performed in a double-blind fashion. The investigator, subjects and all study staff will remain blinded. A non-blinded CHDR staff member (not part of study staff) will receive report of Hb and Ht before dosing and will follow the instruction as described in Figure 1. This individual will be responsible for the dosage adjustments.

Procedure for dosage adjustment:
When the Hb concentration and/or Ht exceeds a certain value or Hb stays below a certain value (see Figure 1) the dose adjustment officer will issue a request for a dosage change for the subject that requires the change. This request will be for the subject that requires the change in treatment but will also be issued to a random placebo subject to preserve the blinding of the study.

18. NeoRecormon detection in urine

Urine will be collected at two predetermined periods. In the second treatment week samples will be taken pre-dose (day 7/8/9), two days later (day 9/10/11), at day 11/12/13/14 before and after the exercise test and pre-dose at day 14/15/16. Additionally, one sample will be taken before and after

the competition (week 9). These samples will be sent to a lab specialized in rHuEPO (NeoRecormon) detection in urine, according to the current protocol of the Dutch Doping Authority.

19. Statistical methodology

All efficacy endpoints will be summarized (mean and standard deviation of the mean, median, minimum and maximum values) by treatment and time, and will also be presented graphically as mean over time, with standard deviation as error bars. Change from baseline results will be utilized in all data summaries. All categorical efficacy endpoints will be summarized by frequencies. The PD endpoints will be analyzed separately by mixed model analyses of variance with treatment, time and treatment by time as fixed effects, with subject and subject by time as random effect, and with the (average) baseline value as covariate for recurring measurements and a one-way ANCOVA with treatment and baseline as covariates for measurements done at baseline and the end of the treatment period.

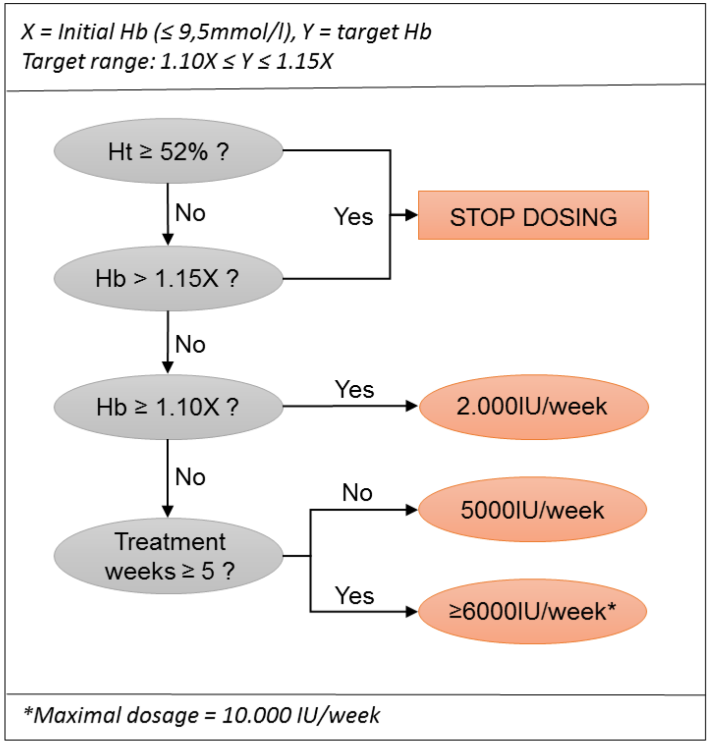

Figure 1. This dosing schedule must be applied before every administration of NeoRecormon/placebo during the 8 week treatment period. Ht = haematocrit, Hb = haemoglobin

Table 1. **Visit and Assessment Schedule.**

	SCR + training	Baseline measurements	Treatment and exercise test weeks*							Competition week			FU
			1, 3, 5, 7		2, 4, 6, 8					9			12
	Day<br>-42 to -3	Day<br>-14 to -1	Day 0/1/2, 14/15/16, 28/29/30, 42/43/44		Ramp exercise tests: Day 11/12/13/14, 25/26/27/28, 39/40/41/42, 53/54/55/56<br><br>Time To Exhaustion exercise test: 46/47/48/49			Day 7/8/9, 21/22/23, 35/36/37, 49/50/51		Weekend after last exercise test			80 +- 7
Time point / Assessment			-6 to -1h	0h	Before ET	During ET	After ET	-6 to -1h	0h	Before competition	During competition	After competition	
Informed consent	X												
Demographics	X												
Inclusion & exclusion criteria	X												
Height and Weight [10]	X				X								X
Medical history	X												
Physical examination	X												X
Concomitant medication	X												
Serology	X												
Blood sample chemistry	X[6]		X		X[9]		X[9]			X		X	X[6]
Blood sample haematology	X		X							X		X	X
Blood sample coagulation	X[3]	X[4]	X	X[8]	X[9]		X[9]			X		X	X[3]
Blood sample biomarker			X[5]		X[11]		X[11]						
Blood sample RNA					X[12]		X[12]						
Urinalysis	X		X							X		X	X
Urine Drug Screen,<br>Breath Alcohol Test	X												
ECG	X		X					X					X
General symptoms	X		X					X		X			X
Vital Signs (HR, BP, Temp)	X		X					X					X
Blood sample haemoglobin and haematocrit "online"			X					X					
Drug (-placebo) administration				X					X				

Ramp exercise test	X[1]	X				X							
TTE exercise test		X				X							
Laser Speckle (LSCI)			X[7]		X[7]		X[7]						
Blood Sample Lactate						X							
Urine doping control				X[2]	X[2]		X[2]	X[2]		X		X	
(S)AE/Con-meds			<----- continuous ----->										
Training			<----- Whole week ----->										

SCR = Screening, ET = Exercise Test, ECG = Electrocardiogram, BP = Blood Pressure, HR = Heart Rate, Temp = temperature, PD = Pharmacodynamics, (S)AE = (Special) Adverse Event, FU = Follow Up, * / means ‘or’

[1] Exercise test training session.
[2] Urine samples for doping control will only be collected in week 2 on day 7/8/9, at 7/8/9 +2 days, 11/12/13/14 and 14/15/16 (meaning post third dose and at day 2, 4 and 7 after that dose)
[3] Short coagulation panel at screening and follow-up, full panel for all other samples
[4] Before and after ramp exercise test
[5] Only on day 0/1/2 and 42/43/44
[6] Full chemistry panel at screening and follow-up, short panel for all other samples
[7] Only at day pre-first-dose and after exercise test on 39/40/41/42 and before exercise test on 46/47/48/49
[8] Only at day 0/1/2 post-dose, 1-3h
[9] Not at Time to Exhaustion test, except for Blood sample chemistry
[10] Height measured at screening only
[11] Before and after exercise test on day 39/40/41/42
[12] Before and after Time to exhaustion test on day 46/47/48/49

Table 2. Exercise parameters in endurance performance.

Abbreviation	Description	Calculations / Characteristics
$V_{O2,\ max}$	Maximal oxygen consumption (ml kg^{-1} min^{-1})	
P_{max}	Maximal power output (W)	1W = 1J/sec
V_E	Respiratory minute ventilation (L/min)	
V_t	Tidal volume (L)	
R_f	Respiratory frequency	
V_{O2}	Oxygen consumption (L/min)	
V_{CO2}	Carbon dioxide production (L/min)	
eqV_{O2}	Ventilatory equivalent for oxygen	V_E/V_{O2}
eqV_{CO2}	Ventilatory equivalent for carbon dioxide	V_E/V_{CO2}
EE	Energy Expenditure (J/sec)	([3.869 xV_{O2}]+[1.195 x V_{CO2}]) x (4.186/60) x 1000
GE	Gross Efficiency (%)	Reflects the percentage of total chemical energy expended that contributes to external work, with the remaining energy lost as heat.<br>(Work rate (J/sec) / Energy Expenditure (J/sec)) x 100%
DE	Delta Efficiency (%)	(Δ Work Production (J/sec)/ Δ Energy Expenditure (J/sec)) x 100%
CE	Cycling economy (W L^{-1} min^{-1})	Can be defined as the submaximal V_{O2} per unit of body weight required to perform a given task.<br>P/V_{O2}
Fat_{max}	The highest absolute fat oxidation	
LT1 = AT = OBLA	Lactate Threshold 1 = Anaerobic Threshold = Onset of Blood Lactate Accumulation	The threshold where blood lactate levels raise 1mmol/L above baseline values.
LT2	Lactate Threshold 2	The highest exercise intensity where lactate levels remain stable.
VT1	Ventilatory Threshold 1	The first increase in V_E that is proportional to the increase in V_{CO2} with no increase in V_{O2}.<br>Increase of end tidal O_2 pressure.
VT2 = RCP	Ventilatory Threshold 2 = Respiratory Compensation Point	Represents itself at a high work.<br>V_E increases far more compared to V_{CO2}.<br>End tidal CO_2 pressure decreases.

1 BACKGROUND AND RATIONALE

1.1 Context

1.1.1 Need for research on doping

A recent report of the Union Cycliste Internationale (UCI) gives an in-depth analysis of doping throughout cycling's history, from 1890 to the present day. The report's final conclusion is that cycling has had, and continues to have, a serious doping problem.[1]
Although it could be argued that administering substances that improve performance is forbidden and nothing more needs to be known about it, research to investigate the effects and safety of doping substances in this population is necessary. There are number of reasons for this. First, it is often unknown if a forbidden substance really enhances performance. If this is not the case the need for administration is strongly diminished. Additionally the adverse effects of such substances are often insufficiently known and athletes may be exposed to risks without being adequately informed about them.

1.1.2 Erythropoietin substances in patients and athletes

Recombinant Human Erythropoietin (rHuEPO) is used to treat patients with anemia resulting from chronic kidney disease.[2] The correction of the anemia results in an increase in exercise capacity in these patients.[2] The treatment immediately attracted the attention of athletes because they assumed that rHuEPO would also improve their exercise performance. Due to this presumption, the use of rHuEPO in athletes became very common. In 1990, the use of rHuEPO was placed on the list of prohibited substances published by the World Anti-Doping Agency (WADA).[3] At the time of the first ban there was no published evidence that rHuEPO would actually improve sports performance.

1.1.3 Examination of the evidence for the ergogenic properties of rHuEPO in cyclists

The evidence for the effect of rHuEPO in well trained athletes is in fact sparse until today. A qualitative systematic review of the available literature was performed in 2012 to examine the evidence for the performance enhancing properties of rHuEPO in cyclists.[4]

The review demonstrated that the characteristics of the study populations differed from the population suspected of rHuEPO abuse. rHuEPO studies often used untrained or moderately trained cyclists.[5] It cannot be assumed that effects found in these studies automatically apply to well-trained cyclists. Most studies used a small number of untrained subjects and the quality of the research was often questionable. In these studies, the main studied effect was the maximal oxygen carrying capacity of blood ($V_{O2,\ max}$) which only has a remote connection to performance in endurance sports, especially in well-trained athletes. This is in line with the knowledge that multiple factors affect performance, in which oxygen carrying capacity of the blood becomes less relevant when other factors become rate-limiting. Endurance performance may be better correlated with submaximal exercise factors. There a number of findings that support this last notion. Firstly, research into training for endurance performance shows that moderately trained athletes are able to improve $V_{O2,max}$ by interval and/or intensive training, whereas these training regimens do not improve $V_{O2,max}$ in well-trained athletes. After initial years of training, these well-trained athletes maintain a plateau in their $V_{O2,max}$, but continue to improve their performance. This shows that other factors have to play an important role in endurance performance.
Secondly, long exercise times during consecutive days, with the finish line as a known end-point (contrary to the ' open end' of time-to-exhaustion tests) makes it crucial for cyclists to distribute their power during a race. This, combined with (team) tactics, the terrain and the effects of drag force, means that cyclists work for only a small amount of time at their peak intensities ($V_{O2,\ max}$), the rest of the time they will work at sub-maximal intensities where submaximal exercise factors are more important.

Finally, adverse events were never studied despite the fact that there is much evidence from patient studies that rHuEPO may cause hypertension and thrombotic events. Uncontrolled use of rHuEPO therefore involves risks for the users' health, irrespective of such a substance being used legally or illegally.

1.1.4 Study objectives

The current study will

- explore the effects of NeoRecormon on cycling performance in well-trained cyclists by
  - performance in *exercise tests*
  - performance in a *competition*
  - measuring markers from the haematological module of the *Athlete Biological Passport*
  - measuring *blood flow*
- evaluate the safety of NeoRecormon in well-trained cyclists.
- evaluate the performance of doping detection methods for NeoRecormon use in well-trained cyclists.

1.2 Non-clinical information

1.2.1 Non-clinical pharmacology

Please refer to the respective Summary of Product Characteristics (SmPC) (see appendix 1) and European Public Assessment Report (EPAR) (see appendix 3).

1.2.2 Non-clinical pharmacokinetics and metabolism

Please refer to the respective SmPC and EPAR (see appendix 1 and 3).

1.2.3 Non-clinical toxicology and safety pharmacology

Please refer to the respective SmPC and EPAR (see appendix 1 and 3).

1.3 Clinical information

1.3.1 Clinical pharmacology

Please refer to the respective SmPC and EPAR (see appendix 1 and 3).

1.3.2 Clinical pharmacokinetics and metabolism

Please refer to the respective SmPC and EPAR (see appendix 1 and 3).

1.3.3 Clinical toxicology and safety pharmacology

Please refer to the respective SmPC and EPAR (see appendix 1 and 3).

1.4 Study rationale

1.4.1 Benefit and risk assessment

NeoRecormon is a registered drug. The safety profile of this compound is known. Because side effects might occur and anaphylactoid reactions were observed in isolated cases (≤1/10.000) (see SmPC, appendix 1), the study drug administrations will be done in the clinic under medical supervision. Subjects will be closely monitored and will only be discharged from the unit if their medical condition allows this.

Subjects will receive a dose of 2000, 5000 or ≥6000IU with a maximum of 10.000IU/week of NeoRecormon once a week for 8 weeks. The dosage depends on haemoglobin (Hb) concentration

and haematocrit (Ht) measured prior to administration. If Ht is ≥52% dosage will be interrupted. If Ht is <52% the dosage depends on the Hb concentration (see Figure 1).
The effects of NeoRecormon used in patients in an autologous blood predonation programme most closely resembles the effects of NeoRecormon in healthy volunteers. For the use of NeoRecormon in an autologous blood predonation programme, the SmPC (see appendix 1) states that the maximum dose should not exceed 1200IU/kg (or 90.000 IU for a 75kg subject) per week for subcutaneous administration. Planned doses of 2000IU, 5000IU or ≥6000IU with a maximum of 10.000IU/week of NeoRecormon will be well below this maximum dose and are therefore considered safe. The risk is considered small and therefore acceptable compared to the scientific benefit.

1.4.2 Medical and regulatory background

The genetically engineered erythropoietin hormone, rHuEPO (in this research NeoRecormon), works in the same way as the natural hormone erythropoietin (EPO). EPO is a (glycoprotein) hormone primarily produced by the kidneys.[8] The kidneys secrete EPO in response to hypoxia in the renal circulation.[8] The secreted EPO binds to the EPO receptor on the erythroid progenitor cell surface and in this way activates intracellular signaling pathways that lead to erythropoiesis in the bone marrow.[8] Erythropoiesis is the proliferation and differentiation of erythroid progenitor cells to erythrocytes (red blood cells).[8] Erythrocytes are responsible for oxygen transport through the blood.[4] Due to the lack of a nucleus and other cellular machinery, erythrocytes are not able to repair themselves.[4] They have a lifespan of approximately 120 days in the circulation after which they are degraded by the spleen (2-3 million every second).[4] To keep the oxygen carrying capacity of the blood at a steady level, constant erythropoiesis is necessary.[4] The concentration of EPO in blood is relatively constant at approximately 5 pmol L^{-1}.[4]

1.4.3 Study population

Forty-eight well-trained male subjects, 18 to 50 years of age who have a Hb level >8.0 mmol/L and ≤ 9.8 mmol/L, a Ht <48% and a maximal power output > 4.0 W/kg will be included.

Previous studies showed that professional cyclists have Hb concentrations between 8.25 and 10.25mmol/l and Ht levels between 39.2 and 48.1%.[9], [10] The normal Hb concentration for males is 8.75 to 11.25 mmol/l.[11;12] The rHuEPO treatment in this study will increase Hb levels of the subjects with 10-15%. Subjects will be excluded during the screening when they have Hb levels >9.8mmol/l, because the normal Hb level (11.25mmol/l) should not be exceeded (9.8 x 1.15 = 11.27 mmol/l).

1.4.4 Study design

This study will be conducted in accordance with guidelines from the International Conference on Harmonization (ICH) on current Good Clinical Practice (GCP) and the ethical principles that have their origins in the Declaration of Helsinki.

In order to investigate the effects and safety of NeoRecormon in well-trained cyclists a randomized, double-blind, placebo-controlled study is most appropriate.

The total study period will be 17 weeks. Within 6 weeks prior to the start of the treatment, subjects will undergo a medical screening. A ramp exercise test on a cycle ergometer will be performed directly after the screening to familiarize with the test and to determine the subject's maximal power output. A second ramp and TTE exercise test will be performed for baseline measurements within 2 weeks prior to the first administration, but after the screening. During the 8 week treatment period subjects will be administered with NeoRecormon or placebo once a week. During the treatment period subjects will follow their usual training program and will perform a ramp exercise test (see appendix 5) every two weeks. In the 7th treatment week the subjects will also perform a TTE

exercise test (see appendix 5). After the treatment period subjects will participate in a competition (see appendix 6). A follow-up visit will be scheduled, 30 days after the last dose.

Please refer to the visit and assessment schedule for a more detailed description of the study design (see Table 1).

1.4.5 Investigational drug and placebo

Investigational drug:	NeoRecormon (Active substance: Epoëtine beta)
Dosage:	2000IU, 5000IU or ≥6000IU with a maximum of 10.000IU/week, depends on Ht percentage and Hb concentration (see Figure 1)
Administration:	Subcutaneously
Placebo:	Saline, 0.90% w/v NaCl.

The investigational drug and its matching placebo are indistinguishable and will be packaged in the same way. Blinding will be accomplished by using the same syringes or by covering the syringes with aluminum foil.

Eligible subjects will be randomized to one of two treatment groups on a 1:1 basis. One group will be treated with placebo and one group with NeoRecormon.

1.4.6 Dosing, safety margin calculations, stopping criteria

Dose selection and adjustments

Please refer to Figure 1 for a detailed dosing schedule.

The review of the data and the decision on the next dose level will be made by a non-blinded CHDR staff member unconnected to the study.

Dosage Rationale

An initial dose of 5000IU/week will be administered because previous studies showed that with this dose the above mentioned target range should be reached.[13-16] If the upper limit of the target range is exceeded the dose will be interrupted until the Hb concentration falls back into the range. At that point, therapy should be restarted at 2000IU/week. If treatment weeks ≥ 5 and Hb < (1.10*Initial Hb) the dose will be adjusted to ≥6000IU/week (maximal 10.000IU/week), so that it is still possible to reach the target range within 8 weeks.

Safety margin calculations

NeoRecormon is a marketed medication. The safety profile of this treatment is well-known (see SmPC (appendix 1) and EPAR (appendix 3)). See chapter 1.4.1 for the benefit and risk assessment.

Subcutaneously administered NeoRecormon has a half-life of 12-28h. There will be one week between subcutaneous administrations of NeoRecormon, which is sufficient time to wash-out treatment from a previous occasion.

Stopping criteria

Dosing will be interrupted if
- Ht ≥ 52%
- Hb exceeds the upper limit of the target range (see Figure 1)
- there's an unacceptable tolerability profile based on the nature, frequency, and intensity of observed AEs

These criteria will be maintained by the non-blinded CHDR staff member unconnected to the study.

1.4.7 Treatment duration

In this study 24 subjects will be injected with NeoRecormon and 24 subjects will be injected with placebo once a week during 8 weeks.

A treatment period of 8 weeks is chosen because this will provide sufficient time to reach the target range. If the target range is not reached after 5 weeks, the dose will be adjusted. In addition, during this treatment period study objectives such as assessment of safety and efficacy can be performed adequately.

1.4.8 Endpoints

Efficacy endpoints

Efficacy will be assessed at the time points indicated in the Visit and Assessment Schedule (Table 1).

Efficacy will be assessed by:
- performance in *exercise tests*
- performance in a *competition*
- measuring markers from the haematological module of the *Athlete Biological Passport*
- measuring *blood flow*

Exercise tests

A review showed that most of the research investigating the effect of rHuEPO in cyclists focused on a parameter for maximal exercise oxygen consumption, $V_{O2,max}$.[4] The review showed that besides this parameter, research should also focus on parameters for sub-maximal exercise. Therefore in this study maximal as well as sub-maximal exercise parameters (see table 2) will be measured during exercise tests. Subjects will perform a ramp exercise test every two weeks during their treatment/training period and a time to exhaustion exercise test in the 7th treatment week to see if the NeoRecormon/placebo treatment has an effect on their performance. Baseline measurements for these exercise tests will be performed one week prior to the treatment period.

Before and during the exercise tests blood will be collected at predetermined stages to determine if certain protein concentrations are influenced by NeoRecormon.
Please refer to appendix 5 for more detailed descriptions of the exercise tests.

Competition

Multiple factors affect cycling performance, especially in the racing conditions seen in official competition. Therefore, in this study the effects of NeoRecormon will also be determined by performance in a competition. The competition will be designed in such a way that it closely resembles real racing conditions. During the competition certain maximal and sub-maximal exercise parameters will be measured. Before and during the competition blood will be collected at predetermined stages to determine if certain protein concentrations are influenced by

NeoRecormon. After the competition urine will be collected for a doping control and for urinalysis for a.o. proteinuria.

Please refer to appendix 6 for a more detailed description of the competition.

Athlete Biological Passport
According to the WADA, the athlete biological passport (ABP) is introduced to establish whether an athlete is manipulating his physiological variables without detecting a particular substance or method. The objective of this testing is to identify athletes in a haematological module and a steroidal module. The haematological module tests for certain markers in the body that identify the enhancement of oxygen transport. The steroidal module collects information on markers for steroid doping and aims to identify endogenous anabolic androgenic steroids. In this study only the haematological module will be conducted because rHuEPO only influences markers of this module.

In this study an ABP will be created to determine the effect of NeoRecormon on markers of the haematological module and in addition to investigate if these markers together are really able to prove that an athlete is manipulating his physiological variables. The ABP will also be administered for safety, because based on Hb concentration and Ht (two of the haematological module markers) a dose will be selected weekly (during the treatment period). Blood to measure the haematological module markers will be collected before NeoRecormon/Placebo administration and before the competition.

Blood flow
LSCI measures:

- basal blood flow
- blood flow upon occlusion-reperfusion
- Blood flow after ‘exercise’

1.4.9 Statistical hypotheses and sample size

1.4.9.1 Power calculation based on $V_{O2,max}$

Null hypothesis:
There’s no difference between the effects of NeoRecormon and placebo on cycling performance parameters.

Based on the results of a previous study a power analysis has been performed.[6] In this study sixteen endurance-trained men (cyclists, runners and triathletes) were assigned randomly to either the rHuEPO- (n=9) or placebo- (n=7) treated groups. Both the participants and the investigators engaged in exercise testing and blood sampling were blind with respect to the group assignment. All athletes had been in regular training for several years preceding the study and continued to train throughout the period of the study but were forbidden to participate in sport events. The load of training was not different between the rHuEPO and placebo groups throughout the 4-weeks treatment period. Moderate doses of rHuEPO or placebo were injected s.c. 3 times a week for 4 weeks in the morning. The two groups also received a daily oral dose of 200mg of iron sulphate during the 4 weeks. $V_{O2,max}$ was evaluated before and after rHuEPO treatment by using a ramp exercise test (30W/min after a 3-min baseline of warm-up at 60W) performed to the limit of tolerance on an electrically braked cycle ergometer that controlled external power output independent of pedal cadence.

The mean $V_{O2,max}$ before treatment was similar in rHuEPO and placebo groups (63.0±1.5 vs 64.8±2.0 ml/min/kg). $V_{O2,max}$ increased significantly after rHuEPO treatment in the rHuEPO group (68.4±1.9 ml/min/kg) and was significantly higher than in the placebo group (64.6±2.0 ml/min/kg, $P<0.05$).

Equation to determine sample size: [17]

$$n = 1 + 2C(s/d)^2$$

n = sample size
α = significance level
1-β = the desired power of the experiment to detect the postulated effect
C = constant dependent on the value of α and β selected (see table 4)
s = standard deviation of the variable
d = magnitude of the difference the investigator wishes to detect

Table 4. Constant C dependent on the value of α and β selected.[17]

		α	
		0.05	0.01
1-β	0.8	7.85	11.68
	0.9	10.51	14.88

Factors from the previous study to calculate sample size: [6]
d = 68.4 (rHuEPO-group) - 64.6 (Placebo group) = 3.8 ml/min/kg
s = 1.95
1 - β = 80%
α = 5%

A sample size of 6 in each group will have a power of 80% to detect a difference in means of 3.8ml/min/kg, assuming that the common standard deviations is 1.95, using a two-tailed t-test with a 0.05 two sided significance level.

A review of the available literature of the research investigating the effect of rHuEPO in cyclists showed that, after initial years of training, well-trained athletes maintain a plateau in their $V_{O2,max}$, but continue to improve their performance further.[4] This indicates that the difference between effects on $V_{O2,max}$ between the NeoRecormon and placebo group in well-trained cyclists will be smaller. The smaller the size of the difference, the larger the sample size must be to detect a significant difference. To detect a difference of 1.5ml/min/kg with a power of 80% a sample size of 22 is needed, assuming that the common standard deviation is 1.95, using a two-tailed t-test with a 0.05 two sided significance level. When taking into account a ±10% attrition rate, 24 subjects are needed in both the NeoRecormon and placebo group.

1.4.9.2 Power calculation based on P_{max}/kg

A better endpoint would be power output per kilogram (P/kg) at a submaximal level, such as 80% of $V_{O2,max}$. Unfortunately no studies have been performed using this endpoint, so the effect of rHuEPO on P/kg at 80% $V_{O2,max}$ is still unknown. The mean P/kg at 80% $V_{O2,max}$ of 11 male professional cyclists however, is 5.2 W/kg with a standard deviation of 0.199.[7] Using a sample size of 22 (including 10% attrition rate) and a two-tailed t-test with a 0.05 two sided significance level a difference of 0.172 W/kg can be detected with a power of 80%. This difference would mean that a professional cyclist weighing 75 kg would go from an average of 390 W at 80% $V_{O2,max}$ to 402.9 W. On a racing bike weighing 9 kg sitting in racing position at 25 degrees Celsius, this would produce a speed of 43.80 km/h and 44.32 km/h respectively (calculated from http://bikecalculator.com). In a flat terrain of 40 km this would result in a finish time of 54 min 48 sec and 54 min 09 sec, a difference of 39 seconds, which is very relevant in a race like to Tour the France.

2 STUDY OBJECTIVES

2.1 Primary objective

To explore the effects of NeoRecormon on cycling performance by

- performance in *exercise tests*
- measuring markers from the haematological module of the *Athlete Biological Passport*
- measuring *blood flow*

in well-trained cyclists.

2.2 Secondary objectives

To explore the effects of NeoRecormon on cycling performance in a competition (road race)

To evaluate the safety of NeoRecormon in well-trained cyclists

To evaluate the performance of doping detection methods for NeoRecormon use in well-trained cyclists

2.3 Exploratory objectives

To explore how a standardized submaximal exercise affects gene expression patterns in well-trained individuals.

To explore the difference in RNA-profiles between individuals treated with rHuEPO and placebo

To identify potential transcripts that can be used as biomarkers for rHuEPO use

To explore correlations between changes in whole blood gene expression patterns observed before and after a submaximal exercise test in individuals and their performance

3 STUDY DESIGN

3.1 Overall study design and plan

This study will explore the effects of NeoRecormon on well-trained cyclists and their cycling performance during exercise tests and in a competition. It will consist of a screening, a training of the ramp exercise tests, 8 treatment visits, an 8 week training program, 7 exercise test visits, a competition and a follow-up visit which are outlined in the study Visit and Assessment Schedule (see Table 1).

The total duration of the study for each subject will be up to 129 days divided as follows:

Study periods**	Days*
Screening + Training ramp exercise test	-42 to -2 days
Ramp + TTE exercise test for baseline measurements	-7 to -1 days
Visit clinical unit: Treatment	0/1/2, 7/8/9, 14/15/16, 21/22/23, 28/29/30, 35/36/37, 42/43/44, 49/50/51
Visit clinical unit: Ramp exercise tests	11/12/13/14, 25/26/27/28, 39/40/41/42, 53/54/55/56
Visit clinical unit: TTE exercise test	46/47/48/49
Training	0 to 55
Competition	57 to 60
Follow-up	80 +- 7

* / means 'or'

** TTE = Time to exhaustion

Dosage is spread over three days because it is logistically very difficult to perform safety measurements in 48 subjects and inject them afterwards with the study drug in one day. The exercise tests are spread over four days for the same reason.

3.1.1 Screening

The screening phase (days -42 to -2) will only be started after full written, verbal and signed informed consent has been obtained, according to CHDR standard operating procedures. The entire screening process will last approximately 2 hours. The screening will be divided into two parts:

Medical screening
- Medical interview
- Physical examination
- 12-lead ECG
- Vital signs (Heart rate, blood pressure)
- Weight
- Height
- Urine Drug Screen (THC, morphine, benzodiazepines, cocaine, amphetamines, methamphetamines, MDMA)
- Alcohol Breath Test
- Blood sampling (haematology, biochemistry, serology, coagulation)
- Urinalysis

Training
- Exercise test performed on an ergometer (see Appendix 5 – Exercise test for a more detailed description)

During screening urine and blood samples will be collected from each patient for analysis as

described in section 7.2.6.

3.1.2 Treatment and exercise tests

The time schedule of study days is provided in general in the Visit and Assessment Schedule (see Table 1). On treatment days subjects will arrive at the clinical unit at a predetermined time. Before dosing standard safety measurements will be performed (see Table 1).

Subjects will perform a ramp exercise test every two weeks during the treatment period. They also will perform a Time To Exhaustion exercise test in the 7th treatment week. Baseline measurements for these tests will be performed 2 weeks prior to the treatment period. During the exercise test maximal and submaximal exercise parameters will be measured. Before and during the exercise tests blood will be collected at predetermined stages.

3.1.3 Training period

During the treatment period (8 weeks) subjects will maintain their usual training programme. They will record their training intensity during this period in a diary. Additionally, subjects bikes will be mounted with a Pioneer power meter that will be used for each training activity to log the trip. This information will be shared with the investigators.

3.1.4 Competition

After the treatment period subjects will participate in a competition. The competition is designed in a way that it closely resembles real racing conditions and complies with the required performance duration. During the competition physiological parameters will be measured. Before and after the competition blood will be collected at predetermined stages.
Please refer to appendix 6 for a detailed description of the competition.

3.1.5 Follow-up

A follow-up visit will be performed 30 days after the last visit, which includes a final physical examination, safety laboratory tests (haematology, chemistry, coagulation and urinalysis) and the measurement of vital signs (see Visit and Assessment Schedule, Table 1). Subjects will also be asked if they believe they were on active or placebo treatment. A description of all procedures and analyses is included in section 7.2.

3.1.6 Urine doping control

Urine will be collected at two predetermined periods. In the second treatment week samples will be taken pre-dose (day 7/8/9), two days later (day 9/10/11), at day 11/12/13/14 before and after the exercise test and pre-dose at day 14/15/16. Additionally, one sample will be taken before and after the competition (week 9). These samples will be sent to a lab (DoCoLab – Ugent, Technologiepark 30, Zwijnaarde) specialized in rHuEPO (NeoRecormon) detection in urine, according to the current protocol of the Dutch Doping Authority.

Please refer to appendix 7 for a detailed description of the urine doping control procedure.

4 STUDY POPULATION

4.1 Subject population

A total of 48 subjects will be enrolled into the study following satisfactory completion of a screening visit where eligibility for the study will be checked. Subjects will be recruited via media advertisements and via advertisements at cycling associations.

4.2 Inclusion criteria

Eligible subjects must meet all of the following inclusion criteria:

- Well-trained (as determined by cycling history and maximal power output >4 W/kg) male subjects, 18 to 50 years old (inclusive);
- Subjects must be healthy / medically stable on the basis of clinical laboratory tests, medical history, vital signs, and 12-lead ECG performed at screening, including exercise ECG.
- Each subject must sign an informed consent form prior to the study. This means the subject understands the purpose of and procedures required for the study.

4.3 Exclusion criteria

Eligible subjects must meet none of the following exclusion criteria:

- Any clinically significant abnormality, as determined by medical history taking and physical examinations, obtained during the screening visit that in the opinion of the investigator would interfere with the study objectives or compromise subject safety.
- Unacceptable known concomitant diagnoses or diseases at baseline, e.g., known cardiovascular, pulmonary, muscle, metabolic or haematological disease, renal or liver dysfunction, ECG or laboratory abnormalities, etc.
- Unacceptable concomitant medications at baseline, e.g., drugs known or likely to interact with the study drugs or study assessments.
- Unacceptable potential cycling performance enhancing medications at baseline, e.g. Erythropoiesis-stimulating agents, Anabolic Androgenic Steroids, Growth Hormone, Insulin, IGF-I and Beta-Adrenergic Agents.
- Blood transfusion in the past three months.
- Loss or donation of blood over 500 mL within three months.
- Participation in a clinical trial within 90 days of screening or more than 4 times in the previous year.
- Known hypersensitivity to the treatment or drugs of the same class, or any of their excipients.
- Any known factor, condition, or disease that might interfere with treatment compliance, study conduct or interpretation of the results such as drug or alcohol dependence or psychiatric disease.
- Positive urine drug test at screening.
- Positive alcohol breath test at screening.
- Haemoglobin (Hb) concentration > 9.8 mmol/l at screening.
- Hb concentration < 8 mmol/l at screening.
- Haematocrit (Ht) ≥ 48% at screening.

- ❖ Being subject to WADA's anti-doping rules, meaning being a member of an official cycling union or other sports union for competition (such as the KNWU) or participating in official competition during the study.
- ❖ Positive results from serology at screening (except for vaccinated subjects or subjects with past but resolved hepatitis)
- ❖ Previous history of fainting, collapse, syncope, orthostatic hypotension, or vasovagal reactions.
- ❖ Any circumstances or conditions, which, in the opinion of the investigator, may affect full participation in the study or compliance with the protocol.

4.4 Concomitant medications

The clinical results obtained so far do not indicate any interaction of NeoRecormon with other medicinal products.

All medications (prescription and over-the-counter [OTC]) taken after study screening until the end of the study will be recorded.

4.4.1 Mandatory concomitant supplementation

Mandatory concomitant supplementation for all 48 subjects:

- ❖ 50mg vitamin C (ascorbic acid) per day
- ❖ 200 mg iron (ferrofumerate) per day

4.4.2 Allowed concomitant medications

Allowed concomitant medications:

- ❖ Paracetamol
- ❖ Other medications are only allowed if they are discussed, approved and clearly documented by the investigator.

4.4.3 Prohibited concomitant medications

Prohibited concomitant medications:

- ❖ All substances (except NeoRecormon during treatment period) that enhance cycling performance are prohibited within 6 months prior to study drug administration and during the course of the study (e.g. Other Erythropoiesis-stimulating agents, Anabolic Androgenic Steroids, Growth Hormone, Insulin, IGF-I and Beta-Adrenergic Agents etc.).

4.5 Lifestyle restrictions

In the interest of the subjects' safety and to facilitate assessment of the treatment effect, the patients participating in this study will be requested to agree to the following restrictions during the study:

- Alcohol will not be allowed from at least 24 hours before screening and before each scheduled visit, and whilst in the study unit until discharge from the study unit.

- During the study subjects are not allowed to be subject to WADA's anti-doping rules, meaning they are for example not allowed to be a member of an official cycling union or other sports union for competition (such as the KNWU). NeoRecormon treated subjects are not allowed to become a member of such a union for 3 months after the last dose.
- Subjects must maintain their usual training programme until the end of the treatment period (week 1-8). They will record their training activity in a diary and using the Pioneer power meter. Directly after the treatment period (week 9) the subjects will participate in a competition (see appendix 6).

4.6 Study drug discontinuation and withdrawal

4.6.1 Study drug interruption or discontinuation

The investigator must temporally interrupt or permanently discontinue the study drug if continued administration of the study drug is believed to be contrary to the best interests of the subject. The interruption or premature discontinuation of study drug might be triggered by an Adverse Event (AE), a diagnostic or therapeutic procedure, an abnormal assessment (e.g., ECG or laboratory abnormalities), or for administrative reasons in particular withdrawal of the subject's consent. The reason for study drug interruption or premature discontinuation must be documented.

4.6.2 Subject withdrawal

Subjects have the right to withdraw from the study at any time for any reason. Should a subject decide to withdraw from the study, all efforts should be made to complete and report the observations, particularly the follow-up examinations, as thoroughly as possible.

4.6.3 Replacement policy

Subjects withdrawing in the first treatment week for reasons other than adverse events or any other tolerability issues with the treatment will be replaced.

5 INVESTIGATIONAL MEDICINAL PRODUCT

5.1 Investigational drug and matching placebo

Study drug or placebo will be administered to the subjects as detailed in Table 1. NeoRecormon will be ordered as available and its matching placebo will be manufactured. The investigational drug and its matching placebo are indistinguishable and will be packaged in the same way. Blinding will be accomplished by using the same syringes or by covering the syringes with aluminum foil.

5.2 Comparative drug

Placebo: Saline, 0.90% w/v NaCl.

5.3 Study drug dosing scheme

Please refer to Figure 1 for a detailed dosing schedule.

5.4 Study drug packaging and labelling

NeoRecormon and placebo will be acquired, packaged and labelled by the LUMC pharmacy in accordance with local regulations. Upon arrival at the pharmacy, the investigational products should be checked for damage and verify proper identity, quantity, integrity of seals and temperature conditions, and report any deviations or product complaints upon discovery. The dispensing of the study drug will be performed by the pharmacy. Study drug will be dispensed for each subject according to the randomization list. Study drug packaging will be overseen by the Leiden University Medical Centre Pharmacy and bearing a label with the identification required by local law, the protocol number, drug identification, and dosage.

All subcutaneously administered drugs and placebos will be indistinguishable and will be packaged in the same way.

The study drug must be stored between 2ºC and 8ºC in a secure, temperature-controlled (refrigerator) area with limited access. The vial must be kept in the outer carton, in order to protect it from light.

For the purpose of ambulatory use, the unreconstituted product may be removed from the refrigerator and be stored at room temperature (not above 25ºC) for one single period of up to 3 days. Leaving the reconstituted solution outside the refrigerator should be limited to the time necessary for preparing the injections.

5.5 Drug accountability

Drug accountability will be maintained by the Leiden University Medical Centre Pharmacy and assessed by maintaining adequate study drug dispensing records.

The investigator is responsible for ensuring that dosing is administered in compliance with the protocol. Delegation of this task must be clearly documented and approved by the investigator. All study drug administration will occur under medical supervision.

5.6 Treatment assignment and blinding

5.6.1 Treatment assignment

Subjects must be randomized in a consecutive order starting with the lowest number. Replacement subjects will be numbered +100, e.g. subject 5 will have subject 105 as replacement, and subject 105 will have subject 205 as replacement, etc.

The randomization code will be generated SAS version 9.1.3 (or a more recent version, if available) by a study-independent, CHDR statistician. To reduce the variability between active and placebo,

block randomisation will be used, with one block of subjects aged 18-34 (inclusive) and another of subjects aged 34-50 (inclusive). The sample size of the blocks will be determined at day -14, when the included subjects have been identified. An attempt will be made to choose the highest block size. The randomization code will be unblinded/broken and made available for data analysis only after study closure, i.e., when the study has been completed, the protocol deviations determined, and the clinical database declared complete, accurate and locked. The randomization code will be kept strictly confidential. Sealed individual randomization codes, per subject and per treatment, will be placed in a sealed envelope containing the and labelled 'emergency decoding envelopes' will be kept in a safe cabinet at CHDR.

5.6.2 Blinding

This study will be performed in a double-blind fashion. The investigator, study staff, subjects and monitor will remain blinded to the treatment until study closure. The investigational drug and its matching placebo are indistinguishable and will be packaged in the same way.

With the exceptions described in this section, the randomization list will not be available to the investigator, study staff, subjects and monitors.

The randomization list will be made available to the pharmacist preparing the study drug, to an individual responsible for dosage adjustments and to statisticians or programmers involved in preparing blinded summaries, graphs and listings to support the dose decisions.

The summaries, graphs and listings provided by the statisticians or programmers will be produced in an area to which other team members do not have access. In addition a single non blinded CHDR staff member unconnected to the study will receive reports on the haemoglobin and haematocrit of the subjects. This individual will be responsible for the dosage adjustments.

The investigator will receive a set of sealed emergency codes to be broken in case of emergency situations. If the identity of the study drug administered needs to be known in order to manage the subject’s condition i.e., in case of a medical emergency or in the case a SUSAR occurs, the treatment emergency code for that subject may be broken and the study drug identified. All such occurrences should be documented in the study file. Treatment emergency codes should not be broken except in emergency situations and, if possible, the investigator should be contacted before the emergency code is opened. Just prior to database lock the unused emergency code labels will be checked and a statement to the effect that all are intact (or not as the case may be) will be made on the database lock form.

6 STUDY ENDPOINTS

6.1 Efficacy endpoints

Efficacy will be assessed at the time points indicated in the Visit and Assessment Schedule (Table 1).

Efficacy will be assessed by:
- performance in *exercise tests*
- performance in a *competition*
- measuring markers from the haematological module of the *Athlete Biological Passport*
- measuring *blood flow*

Exercise tests
All subjects will breathe during the exercise test through a facemask that will be connected to an oxymeter to collect inspired and expired gasses for analyzing:
- Oxygen consumption, V_{O2} (L/min)
- Carbon dioxide production, V_{CO2} (L/min)
- Respiratory minute ventilation, V_E (L/min)
- Tidal volume, V_t (L)
- Respiratory frequency, R_f
- Maximal oxygen consumption, $V_{O2,max}$ (ml kg^{-1} min^{-1})

During the exercise tests blood will be collected at predetermined stages to measure:
- Lactate levels
- Tissue plasminogen activator
- Creatinine phosphokinase
- C-reactive protein levels

V_{O2} and V_{CO2} will be used to calculate:
- Ventilatory equivalent for oxygen (V_E/V_{O2}), eqV_{O2}
- Ventilatory equivalent for carbon dioxide (V_E/V_{CO2}), eqV_{CO2}

these values will be used to determine:
- Ventilatory threshold 1, VT1
- Ventilatory threshold 2, VT2

Physiological parameters that will be determined at VT1 and VT2:
- Oxygen consumption, V_{O2} (L/min)
- Oxygen consumption per kg, V_{O2} (L/min/kg)
- Percentage of maximal oxygen consumption, $\%V_{O2max}$ (L/min)
- Power output, P (J/s)
- Power output per kg, P (J/s/kg)

Physiological parameters that will be determined at maximal effort:
- Maximal oxygen consumption, V_{O2max} (L/min)
- Maximal oxygen consumption per kg, V_{O2max} (L/min/kg)
- Maximal power output, P_{max} (J/s)
- Maximal power output per kg, P_{max} (J/s/kg)
- Lactate values

Other determinations:
- Lactate threshold 1, LT1
- Lactate threshold 2, LT2
- Cycling economy, CE (W L^{-1} min^{-1})
- Gross efficiency, GE (%)

- Heart rate (bpm)
- Systolic blood pressure (mmHg)
- Diastolic blood pressure (mmHg)

Competition
Exercise parameters that will be measured and calculated during the competition:

- Result in the race
- Total time of the race
- Power (W)
- Heart rate (bpm)
- Systolic blood pressure (mmHg)
- Diastolic blood pressure (mmHg)

Before and after the competition blood will be collected to determine if certain protein concentrations are influenced by NeoRecormon such as:

- Lactate
- Tissue plasminogen activator
- Creatinine phosphokinase
- C-reactive protein
- Coagulation and activation markers (D-Dimer, F1+2, p-Selectin, e-Selectin, ThromboxaneB2, bTG, PF4, thrombomodulin)

In urine:

- Proteinuria

Athlete Biological Passport
The following Markers are considered within the ABP Haematological Module:

- Haematocrit (Ht)
- Haemoglobin (Hb)
- Red blood cell (erythrocyte) count (RBC)
- Red blood cell mass (RCM)
- Reticulocytes percentage (RET%)
- Reticulocyte count (RET#)
- Mean corpuscular volume (MCV)
- Mean corpuscular haemoglobin (MCH)
- Mean corpuscular haemoglobin concentration (MCHC)
- Red cell distribution width (standard deviation) (RDW-SD)
- Immature reticulocyte fraction (IRF)

Further calculated markers specific to the Haematological Module include OFF-hr Score (OFFS), which is a combination of Hb and RET%[18], and Abnormal Blood Profile Score (ABPS), which is a combination of Ht, Hb, RBC, RET%, MCV, MCH, and MCHC[19].

Blood flow
LSCI measures:

- basal blood flow
- blood flow upon occlusion-reperfusion
- Blood flow after 'exercise'

6.2 Safety endpoints

Safety measurements before NeoRecormon/placebo administration
Safety will be assessed by:

- Monitoring vital signs
  - Pulse Rate (bpm)
  - Systolic blood pressure (mmHg)
  - Diastolic blood pressure (mmHg)
  - Temperature measurements (ºC)
- Electrocardiogram (ECG) (at rest)
  - Heart Rate (HR) (bpm), PR, QRS, QT, QTcB
- Clinical Laboratory Assessments
  - Haematology

    Ht must be <52%. If Ht level is ≥52%, therapy should be interrupted until the Ht level begins to fall.

    *Hb must be below 1.15*Initial Hb (see Figure 1). If Hb is above that level, dosage should be interrupted until the Hb concentration falls back into the range. Then the dosage should be restarted at 2000IU/week of the previous dose.*
  - Chemistry
  - Urinalysis
  - Coagulation

In addition a single non blinded CHDR staff member unconnected to the study will receive reports on the Hb and Ht of the subjects. This individual will be responsible for the dosage adjustments.

Procedure for dosage adjustment:
When the Hb and/or Ht exceeds a certain value (see 5.3) the dose adjustment officer will issue a request for a dosage change for the subject that requires the change. This request will be for the subject that requires the change in treatment but will also be issued to a random placebo subject to preserve the blinding of the study.

Additional safety measures

A single non-blinded CHDR staff member unconnected to the study will receive reports on Hb concentration and Ht of the subjects. This individual will be responsible for the dosage adjustments. If Ht exceeds 52% in a subject, dosage will be stopped and if necessary, ±0.5L blood will be collected from that subject to rearrange a normal Ht percentage. This subject will then be excluded from the study.

(Serious) Adverse Events ((S)AEs) will be collected throughout the study.

6.3 Exploratory endpoints

- RNA expression levels in venous blood before and after a submaximal exercise test

7 STUDY ASSESSMENTS

See Table 1 for the time points of the assessments.

7.1 Exercise-specific screening assessments

7.1.1 Exercise test

For the exercise test at screening, a ramp protocol will be followed until exhaustion. The details of the test will be described in the exercise manual.

7.1.2 Questionnaire

Please refer to appendix 4.

7.2 Safety and tolerability assessments

The definitions, reporting and follow-up of AEs and SAEs are described in section 8.

7.2.1 Specific safety assessments

A single non-blinded CHDR staff member unconnected to the study will receive reports on the haemoglobin (Hb) concentration and haematocrit (Ht) of the subjects. This individual will be responsible for the dosage adjustments.
If Ht exceeds 52% in a subject, dosage will be stopped and if necessary, ±0.5L blood will be collected from that subject to correct the Ht percentage to a normal level. This subject will then be excluded from the study.

7.2.2 Vital signs

Evaluations of systolic and diastolic blood pressure, pulse rate and temperature will be performed throughout the study. Pulse and blood pressure will be taken after 5 minutes in the supine position. Automated oscillometric blood pressures will be measured using a Dash 3000, Dash 4000, Dynamap 400 or Dynamap ProCare 400. Additionally, the pulse rate data provided by the pulse oximeter attached to the monitor.

7.2.3 Weight and height

Weight (kg) will be recorded at screening, before each exercise test and the follow-up visit or upon early termination. Height (cm) will be recorded and body mass index (BMI) calculated at screening.

7.2.4 Physical examination

Physical examination (i.e., inspection, percussion, palpation and auscultation) is performed during the course of the study. Clinically relevant findings that are present prior to study drug initiation must be recorded with the subject's Medical History. Clinically relevant findings found after study drug initiation and meeting the definition of an AE (new AE or worsening of previously existing condition) must be recorded.

7.2.5 Electrocardiography

12-lead electrocardiographs (ECGs) will be obtained during the course of the study using Marquette 800/5500 or Dash3000 and stored using the MUSE Cardiology Information System. The investigator will assess the ECG recording as 'normal', 'abnormal - not clinically significant', or 'abnormal - clinically significant' and include a description of the abnormality as required. The ECG parameters assessed will include heart rate, PR, QRS, QT, and QTcB (calculated using Bazett's method).

7.2.6 Laboratory assessments

Laboratory parameters

Blood and other biological samples will be collected for the following clinical laboratory tests:

Lab	Tests	Collection & Analysis
Haematology	Haemoglobin [including Mean Corpuscular volume (MCV), Mean corpuscular	4 mL of venous blood in a BD Vacutainer® K2EDTA tube.

	haemoglobin (MCH), Mean corpuscular haemoglobin concentration (MCHC)], haematocrit, red cell count (RBC), Red cell distribution width (standard deviation) (RDW-SD), reticulocyte count (RET#), reticulocytes percentage (RET%), immature reticulocyte fraction (IRF), total white cell count (WBC), leukocyte differential count and Platelet count. Differential blood count, including: basophils, eosinophils, neutrophils, lymphocytes, and monocytes.	Samples will be analysed by the Central Clinical Hematology Laboratory (CKHL) of Leiden University Medical Center.
Chemistry and electrolytes	Sodium, potassium, calcium, inorganic phosphate, total protein, albumin, triglycerides, blood urea nitrogen (BUN), creatinine, ferritin, creatinine phosphokinase (CPK)*, uric acid, total bilirubin[1], alkaline phosphatase, AST, ALT, gamma-GT, LDH, C-reactive protein*.	8.5 mL or 2 mL of venous blood in a BD Vacutainer® SST Gel and Clot Activator tube. Samples will be analysed by the Central Clinical Chemistry Laboratory (CKCL) of Leiden University Medical Center.
Coagulation	APTT*, PT*, fibrinogen, D-dimer, F1+2, bTG, PF4, P-selectin, E-selectin, thrombomodulin, TXB2, TNF-alfa	2.7 mL of venous blood in a 3.2% citrate BD Vacutainer and 4 mL in CTAD. Samples will be analysed by Good Biomarker Sciences (GBS) in Leiden and labs to be determined.
RNA expression	RNA profiles	2.5 mL of venous blood in PAXgene vacuum tubes using a blood collection system analysed by Leiden Genome Technology Center (LUMC)
Serology	HIV1 and HIV2 antibodies, Hepatitis B antigen and Hepatitis C antibodies	5 mL of venous blood in a BD Vacutainer® SST Gel and Clot Activator tube. Samples will be analysed by the Central Clinical Microbiology Laboratory (CKML) of the Leiden University Medical Center.
Urinalysis	Leucocytes, blood, nitrite, protein, urobilinogen, bilirubin, pH, specific gravity, ketones, glucose. If there is a clinically significant positive result, urine will be sent to the CKCL for microscopy and/or culture.	A midstream, clean-catch urine specimen will be analysed by dipstick (Multistix® 10 SG, Siemens Healthcare Diagnostics, Frimley, UK).
Alcohol	Alcohol Breath Test	The hand-held Alco-Sensor IV meter (Honac, Apeldoorn, the Netherlands) will be used to measure the breath ethanol concentrations.
Urine drug screen	Cocaine, amphetamines, opiates (morphine), benzodiazepines, methamphetamine, MDMA and cannabinoids.	A urine specimen will be analysed at CHDR by test kit (InstAlert, Innovacon, San Diego, USA).

[1]Conjugated bilirubin will be reported only when total bilirubin is outside the reference range.
*Part of the short chemistry/coagulation panel

The results of the haematology assessment will be used for the safety report and for the athlete's biological passport.

7.3 Efficacy assessments

7.3.1 Exercise tests

Please refer to Appendix 5 – Exercise test for a detailed description of the exercise tests.

7.3.2 Competition

Please refer to Appendix 6 – Competition for a detailed description of the competition.

7.3.3 Athlete biological passport

This assessment has already been performed for safety measurements. The results will be used for the safety report and for the athlete's biological passport.

Further calculated markers specific to the Haematological Module of the ABP include OFF-hr Score (OFFS), which is a combination of Hb and RET%[17], and Abnormal Blood Profile Score (ABPS), which is a combination of Ht, Hb, RBC, RET%, MCV, MCH, and MCHC[18].

7.3.4 Blood flow

LSCI measures:

- basal blood flow
- blood flow upon occlusion-reperfusion
- Blood flow after 'exercise'

Forearm blood flow will be measured by non-invasivce laser speckle contrast imaging (LSCI; PeriCam PSI System, Perimed). At baseline and after 7 weeks of dosing, the flow will be measured at rest, during a five minute occlusion of the brachial artery and during reperfusion after the occlusion. Blood flow will be measured after exercise at 6 weeks. Procedures for LSCI, brachial artery occlusion-reperfusion are described in SOPs. Briefly, the subject will be seated with the left arm placed on a (table) rest. A suitable area of the volar side of the forearm will be identified. This area will be 'illuminated' by the laser and the response signal will be captured.

7.4 NeoRecormon detection assessments

7.4.1 Urine doping screen

Please refer to Appendix 7 – Urine doping control procedure for a detailed description of the urine doping control procedure.

7.5 RNA expression levels

In general, genes react to internal or external stimuli by becoming more active, less active or showing no response. Training stimuli induce stress resulting in changes in gene expression patterns. Gene expression (RNA-) profiles made from whole venous blood taken before and after physical exercise differ between non-trained and trained individuals. This suggests that adaptation to training can be assessed using gene expression analysis. Changes in gene expression patterns can then be used to monitor training effects. It is unclear how adaptation to training with or without use of rHuEPO will change gene expression profiles in well-trained individuals. We hypothesize that the changes in these patterns observed in samples taken before and after an exercise test are correlated with the performance and health status of individual athletes. The measurements of maximal and submaximal exercise parameters and clinical laboratory assessments of the participants in this study will be combined with gene expression profile data to investigate if specific changes in the acute response to a submaximal exercise test are correlated with better endurance

performance or other parameters. Comparisons of these profiles in time are expected to reveal the effects of specific types of training and changes in health status.

RNA profiles from whole venous blood samples will be determined using next generation sequencing. Samples for RNA-profiling will be collected around a submaximal intensity test (45 minutes, starting at 80% Power from the baseline maximal exercise test allowing changes to the power at any time by the subject himself if he wishes). Blood samples (2.5 ml) for RNA analysis will be drawn in PAXgene vacuum tubes at 2 time points, before and directly after the test. RNA-profiles made from both time points of each individual will be compared to identify changes in gene expression due to the submaximal intensity test. Profiles from the same time points will also be compared to determine interindividual variation and possible EPO treatment effects.

Expression levels of the different transcripts in the RNA samples will be determined as digital counts using standard bioinformatics pipelines. Cluster analysis and principal component analysis will be performed to determine the relationships between the samples. In this way it will be possible to determine if:

- The expression patterns differ at the time points before and after submaximal exercise test between individuals and between groups.
- rHuEPO treatment does influence gene expression levels.

Multivariate statistics will be applied to analyse and interpret complete RNA-profiles. The leukocyte composition of the original blood samples is correlated with cell-specific transcripts. Therefore, this can be derived from RNA-profiles allowing their correction for this effect.

Comparison with other parameters measured during the study may indicate which changes in gene expression patterns of individuals are correlated with their performance.

For all subjects RNA samples will be collected for potential analyses of gene expression. Instructions for collection, processing, handling and shipment of the samples will be outlined in the laboratory manual. Samples will be archived according to local administration regulations.

Analysis will be performed outside the scope of the main study and reported separately.

7.6 Sequence of assessments and time windows

Assessments will be performed in the following order, where possible: physical examination, weight and height, ECG, vital signs, blood samples, dosing.

The deviations of actual time points from the expected time points will be within ten percent, calculated from the zero point (time of drug administration) or the last relevant activity. Deviations of more than 10% will be explained in a note. Pre-dose assessments are given in indicative expected times.

7.7 Total blood volume

Sample	Samples taken		Sample Volume*			Volume	
Haematology	8	x	4	mL	=	32	mL
Chemistry	2	x	8.5	mL	=	17	mL
Chemistry short	16	x	2.5	mL	=	40	mL
Serology	1	x	5	mL	=	5	mL
Coagulation	17	x	6.2	mL	=	105.4	mL
Coagulation short	2	x	4.5	mL	=	9	mL
RNA expression	2	x	2.5	mL	=	5	mL
Biomarker	4	x	4	mL	=	16	mL
Hb/Hct (1mL tube)	8	x	1	mL	=	8	mL
Lactate (Drop)	51	x	0.8	mL	=	40.8	mL
* inclusive discarded volume			**Total blood volume/subject**			**278.2**	**mL**

8 SAFETY REPORTING

8.1 Definitions of adverse events

An Adverse Event (AE) is any untoward medical occurrence in a subject who is participating in a clinical study performed. The adverse event does not necessarily have to follow the administration of a study drug, or to have a causal relationship with the study drug. An adverse event can therefore be any unfavourable and unintended sign (including an abnormal laboratory or vital sign finding), symptom, or disease temporally associated with the study participation, whether or not it is related to the study drug.

8.1.1 Intensity of adverse events

The intensity of clinical AEs is graded three-point scale as defined below:

- Mild: discomfort noticed but no disruption of normal daily activity;
- Moderate: discomfort sufficient to reduce or affect normal daily activity;
- Severe: inability to work or perform daily activity.

8.1.2 Relationship to study drug

For each adverse event the relationship to drug as judged by the investigator:

- Probable;
- Possible;
- Unlikely;
- Unrelated.

8.1.3 Chronicity of adverse events

The chronicity of the event will be classified by the investigator on a three-item scale as defined below:

- Single occasion: single event with limited duration;
- Intermittent: several episodes of an event, each of limited duration;
- Persistent: event which remained indefinitely.

8.1.4 Action

Eventual actions taken will be recorded.

8.1.5 Serious adverse events

A Serious Adverse Event (SAE) is defined by the International Conference on Harmonization (ICH) guidelines as any AE fulfilling at least one of the following criteria:

- Is fatal
- Is life-threatening
- Is disabling
- Requires or prolongs in-patient hospitalisation
- Causes congenital anomaly

will be described as a SAE. Important medical events that may not be immediately life threatening or result in death or hospitalisation may be considered a serious adverse event when, based on appropriate medical judgement, they may jeopardise the subject or may require medical or surgical intervention to prevent one of the outcomes listed above.

8.1.6 Suspected unexpected serious adverse reactions

A SUSAR (Suspected Unexpected Serious Adverse Reaction) is a serious adverse event that is unexpected, (nature or severity of which is not consistent with the applicable product information (e.g., investigator's brochure for an unauthorised investigational product or summary of product characteristics for an authorised product)) and suspected (a reasonable possibility of causal relationship with investigational drug).

8.1.7 Reporting of serious adverse events

SAEs and SUSAR's will be reported according to the following procedure.

All SUSARs and SAEs must be reported to the investigator.

In case of multi-centre studies of which a CHDR employee is the responsible investigator, the sub-investigators at sites outside CHDR must notify the investigator by telephone and in writing preferably using a standard form (e.g. CIOMS for SUSARs).

The investigator must report all SAEs and SUSAR's to the EC that approved the study, in writing as soon as practical, but at least within 15 days. Fatal and life-threatening suspected SUSAR's should be reported within 7 calendar days, with another 8 days for completion of the report.

The investigator must report all SUSAR's to the CA, in writing as soon as practical, but at least within 15 days. Fatal and life-threatening suspected SUSAR's should be reported within 7 calendar days, with another 8 days for completion of the report. SAE's do not have to be reported to the CA.

The investigator must furthermore report all SUSAR's to EMA's EudraVigilance database within 15 days. Fatal and life-threatening suspected SUSAR's should be reported within 7 calendar days, with another 8 days for completion of the report.

The investigator can prepare additional reports for other authorities (e.g. FDA).

All SAEs and SUSAR's must be reported on the Toetsing Online website[2]. Fatal and life-threatening SAEs and SUSARs must be reported within 7 calendar days. All other SAEs must be reported within 15 days. By reporting on the Toetsing Online website the EC that approved the study will also be informed.

8.1.8 Follow-up of adverse events

All adverse events will be followed until they have abated, returned to baseline status or until a stable situation has been reached. Depending on the event, follow up may require additional tests or medical procedures as indicated, and/or referral to the general physician or a medical specialist.

8.2 Section 10 WMO event

In accordance to section 10, subsection 1, of the WMO, the investigator will inform the subjects and the EC if anything occurs, on the basis of which it appears that the disadvantages of participation may be significantly greater than was foreseen in the research proposal. The study will be suspended pending further review by the EC, except insofar as suspension would jeopardise the subjects' health. The investigator will ensure that all subjects are kept informed.

8.3 Annual safety report or development safety update report

In addition to the expedited reporting of SUSARs, the investigator will submit, once a year throughout the clinical trial, a safety report to the EC, CA, MEB and CAs of the concerned Member States.

---

[2] https://www.toetsingonline.nl/

This safety report consists of:

- a list of all suspected (unexpected or expected) serious adverse reactions, along with an aggregated summary table of all reported serious adverse reactions, ordered by organ system, per study;
- a report concerning the safety of the subjects, consisting of a complete safety analysis and an evaluation of the balance between the efficacy and the harmfulness of the medicine under investigation.

9 STATISTICAL METHODOLOGY AND ANALYSES

9.1 Statistical analysis plan

All safety and statistical programming is conducted with SAS 9.4 for Windows (SAS Institute Inc., Cary, NC, USA). PK variable programming is conducted with R 2.12.0 for Windows (R Foundation for Statistical Computing/R Development Core Team, Vienna, Austria, 2010.

A Statistical Analysis Plan (SAP) will be written and finalized before the study closure, i.e., database closure and unblinding of the randomization code. The SAP will provide full details of the analyses, the data displays and the algorithms to be used for data derivations.

The SAP will include the definition of major and minor protocol deviations and the link of major protocol deviations to the analysis sets.

9.2 Protocol violations/deviations

Protocol deviations will be identified based on conditions related to the categories below:

- Protocol entry criteria
- Forbidden concomitant medications
- Missing evaluations for relevant endpoints
- Other protocol deviations occurring during study conduct.

Major protocol deviations will be identified before the study closure, and listed where appropriate.

9.3 Power calculation

Please refer to section 1.4.9.

9.4 Missing, unused and spurious data

All missing or incomplete safety and efficacy data, including dates and times, are treated as such. Missing test results or assessments will not be imputed. Missing efficacy data, indicated as 'M' in the data listing, will be estimated within the statistical mixed model using SAS PROC MIXED.

For graphical and summary purposes efficacy and safety values below the limit of quantification will be set to half (½) of the limit of quantification. For analysis no undetermined values will be replaced. The handling of missing, unused and spurious data will be documented in the study report.

9.5 Analysis sets

Data of all subjects participating in the study will be included in the analyses if the data can meaningfully contribute to the objectives of the study.

9.5.1 Safety set

The safety population will be defined as all subjects who were validated (randomised) and received at least 1 dose of study treatment.

9.5.2 Efficacy analysis set

The analysis population for efficacy is defined as all subjects who were validated (randomised), received at least one dose of study treatment, and have at least one post-baseline assessment of the parameter being analysed.

9.6 Subject disposition

The following subject data will be summarized by treatment and overall:

- Number and percentage of subjects enrolled in each analysis set for all randomized subjects;
- Number and percentage of subjects who completed the study or prematurely discontinued from the investigational period by reasons for discontinuation, to be tabulated for each analysis set.

Subject disposition will be listed.

9.7 Baseline parameters and concomitant medications

9.7.1 Demographics and baseline variables

Continuous demographic variables (e.g., age, height, weight, BMI) will be summarized by descriptive statistics (n, mean, SD, median, Min, Max).Qualitative demographic characteristics (sex, race/ethnicity) will be summarized by counts and percentages.

The results of the sport activities questionnaire at screening will only be listed.

9.7.2 Medical history

Medical history will only be listed.

9.7.3 Concomitant Medications

Previous and concomitant medications will be coded according to the World Health Organization (WHO) drug code and the anatomical therapeutic chemical (ATC) class code.

All concomitant medications will be displayed in a listing.

9.7.4 Treatment compliance/exposure

Exposure to study treatment is described in terms of duration of treatment and average infusion rate. The average infusion rate (mL/hr) is summarized by mean, SD, median, Q1, Q3, Min, Max.

9.8 Safety and tolerability endpoints

The safety set is used to perform all safety analyses.
Baseline is defined as the last value prior to dosing. Change from baseline will be calculated for all continuous safety parameters.

9.8.1 Adverse events

The AE coding dictionary for this study will be Medical Dictionary for Regulatory Activities (MedDRA). It will be used to summarize AEs by primary system organ class (SOC) and preferred term (PT).
All adverse events will be displayed in listings.

A treatment-emergent adverse event (TEAE) is defined as an adverse event observed after starting administration of the specific treatment, and prior to the start of another treatment, if any OR up to 5 days (96 hours) after study drug administration. If a subject experiences an event both prior to and after starting administration of a treatment, the event will be considered a TEAE (of the treatment) only if it has worsened in severity (i.e., it is reported with a new start date) after starting administration of the specific treatment, and prior to the start of another treatment, if any. All TEAEs collected during the investigational period will be summarized.

The number of and/or the number of subjects with treatment emergent AEs will be summarized by:

1. treatment, MedDRA SOC and PT;
2. treatment, MedDRA SOC, PT and severity;
3. treatment, MedDRA SOC, PT and drug relatedness.

9.8.2 Vital signs

At each time point, absolute values and change from baseline of supine BP and HR will be summarized with n, mean, SD, SEM, median, Min, and Max values. The number of available observations and out-of-range values (absolute and in percentage) will be presented. Values outside the reference range will be flagged in the listing. ‘H’ and ‘L’, denoting values above or below the investigator reference range (when present), will flag out-of-range results.

9.8.3 ECG

At each time point, absolute values and change from baseline of ECG numeric variables will be summarized with n, mean, SD, SEM, median, Min, and Max values. The number of available observations and out-of-range values (absolute and in percentage) will be presented. Values outside the investigator’s normal range will be flagged in the listing. ‘H’ and ‘L’, denoting values above or below the investigator reference range (when present), will flag out-of-range results.

9.8.4 Clinical laboratory tests

At each time point, absolute values and change from baseline of clinical laboratory variables will be summarized with n, mean, SD, SEM, median, Min, and Max values. The number of available observations and out-of-range values (absolute and in percentage) will be presented. All laboratory data (including re-check values if present) will be listed chronologically. ‘H’ and ‘L’, denoting values above or below the investigator reference range (when present), will flag out-of-range results.

9.9 Efficacy endpoints

9.9.1 Efficacy

The final analysis will be preceded by a blind data review which consists of individual graphs per visit by time of all efficacy measurements by time. The graphs will be used to detect outliers and measurements unsuitable for analysis. The efficacy parameters will be listed by treatment, subject, visit and time. Individual graphs by time will be generated.

All measured PD endpoints will be summarised (n, mean, SD, SEM, median, Min and Max values) by treatment and time, and will also be presented graphically as mean over time, with standard deviation as error bars. All efficacy endpoints will be summarised (mean, SD, SEM, median, Min and Max values) by treatment, and will also be presented graphically as mean in a bargraph, with standard deviation as error bars.

Parameters will initially be analyzed without transformation, but if the data suggest otherwise, log-transformation may be applied. Log-transformed parameters will be back-transformed after analysis where the results may be interpreted as percentage change.

To establish whether significant treatment effects can be detected on the measured efficacy parameters, each parameter that is measured repeatedly will be analyzed with a mixed model analysis of covariance (ANCOVA) with treatment, time and treatment by time as fixed factors and subject, subject by treatment and subject by time as random factors and the (average) baseline measurement as covariate. Parameters that are only measured at baseline and at the end of the treatment phase will be analysed with a one-way ANCOVA with treatment and baseline as covariate.

Single measured efficacy parameters will be analyzed with an unpaired t-test.

The Kenward-Roger approximation will be used to estimate denominator degrees of freedom and model parameters will be estimated using the restricted maximum likelihood method.

The general treatment effect and specific contrasts will be reported with the estimated difference and the 95% confidence interval, the least square mean estimates and the p-value. Graphs of the Least Squares Means(LSM) estimates over time by treatment will be presented with 95% confidence intervals as error bars, as well as change from baseline LSM estimates.

The following contrasts will be calculated within the model:

- NeoRecormon - Placebo

9.9.2 Inferential methods

Null hypothesis:
There’s no difference between the effects of NeoRecormon and placebo on cycling performance

parameters.

9.10 Exploratory analyses and deviations

Exploratory data-driven analyses can be performed with the caveat that any statistical inference will not have any confirmatory value. Deviations from the original statistical plan will be documented in the clinical study report.

9.11 Interim analyses

No interim analysis is planned.

10 GOOD CLINICAL PRACTICE, ETHICS AND ADMINISTRATIVE PROCEDURES

10.1 Good clinical practice

10.1.1 Ethics and good clinical practice

The investigator will ensure that this study is conducted in full compliance with the protocol, the principles of the Declaration of Helsinki, ICH GCP guidelines, and with the laws and regulations of the country in which the clinical research is conducted.

10.1.2 Ethics committee / institutional review board

The investigator will submit this protocol and any related documents to an Ethics Committee (EC) and the Competent Authority (CA). Approval from the EC and the statement of no objection from the CA must be obtained before starting the study, and should be documented in a dated letter/email to the investigator, clearly identifying the trial, the documents reviewed and the date of approval. A list of EC members must be provided, including the functions of these members. If study staff were present, it must be clear that none of these persons voted.

Modifications made to the protocol after receipt of the EC approval must also be submitted as amendments by the investigator to the EC in accordance with local procedures and regulations.

10.1.3 Informed consent

It is the responsibility of the investigator to obtain written informed consent from each individual participating in this study after adequate explanation of the aims, methods, objectives and potential hazards of the study. The investigator must also explain to the subjects that they are completely free to refuse to enter the study or to withdraw from it at any time for any reason.

The Informed Consent and Subject Information will be provided in dutch.

10.1.4 Insurance

The investigator has a liability insurance which is in accordance with article 7, subsection 6 of the WMO.
The investigator has an insurance which is in accordance with the legal requirements in the Netherlands (Article 7 WMO and the Measure regarding Compulsory Insurance for Clinical Research in Humans of 23rd June 2003). This insurance provides cover for damage to research subjects through injury or death caused by the study.

- € 650,000.- (i.e., six hundred and fifty thousand Euro) for death or injury for each subject who participates in the Research;
- € 5,000,000.- (i.e., five million Euro) for death or injury for all subjects who participate in the Research;
- € 7,500,000.- (i.e., seven million five hundred thousand Euro) for the total damage incurred by the organisation for all damage disclosed by scientific research for the Sponsor as ‘verrichter’ in the meaning of said Act in each year of insurance coverage.

The insurance applies to the damage that becomes apparent during the study or within 4 years after the end of the study.

10.2 Study funding

CHDR is the sponsor of the study and is funding the study.

10.3 Data handling and record keeping

10.3.1 Data collection

Data will be recorded on paper and/or electronic data collection forms and will be entered after quality control in a Promasys database for subsequent tabulation and statistical analysis. The data will be handled confidentially and if possible anonymously.

A Subject Screening and Enrolment Log will be completed for all eligible or non-eligible subjects with the reasons for exclusion.

10.3.2 Database management and quality control

A quality control check will be done by CHDR staff using data entry progress checks and database listings (blind data review). Errors with obvious corrections will be corrected before database lock. Results of computer tests and electronically captured questionnaires, clinical laboratory and efficacy analyses will be sent electronically to CHDR and loaded into the database.
After the database has been declared complete and accurate, the database will be locked. Any changes to the database after that time can only be made by joint written agreement between the investigator and the statistician.

10.4 Access to source data and documents

All study data will be handled confidentially. The investigator will retain the originals of all source documents generated at CHDR for a period of 2 years after the report of the study has been finalised, after which all study-related documents will be archived (at a minimum) on micro-film which will be kept according to GCP regulations.
The investigator will permit trial-related monitoring, audits, EC review and regulatory inspections, providing direct access to source data and documents.

10.5 Quality control and quality assurance

This study will be conducted according to applicable Standard Operating Procedures (SOPs). Quality assurance will be performed under the responsibility of CHDR's Quality Assurance manager.

10.5.1 Monitoring

An initiation visit will be performed before the first subject is included. Monitoring visits and contacts will occur at regular intervals thereafter, according to a frequency defined in the study-specific monitoring plan. A close-out visit will be performed after study closure.

10.6 Protocol amendments

Any change to a protocol has to be considered as an amendment.

10.6.1 Non-substantial amendment

Administrative or logistical minor changes require a non-substantial amendment. Such changes include but are not limited to changes in study staff or contact details or minor changes in the packaging or labelling of study drug. Non-substantial amendments will be approved (signed) by the investigator(s) and will be recorded and filed by the investigator but will not be notified to the EC and the CA.

The implementation of a non-substantial amendment can be done without notification to the appropriate EC or CA. It does not require their approval.

The following amendments will be regarded as non-substantial:

- change in timing of the samples;
- changes in assay-type and / or institution where an assay will be performed, provided that validated assays will be used;
- editorial changes to the volunteer information sheets;
- determination of additional parameters in already collected materials, which are in agreement with the study objectives and do not provide prognostic or genetic information;
- other statistical analyses than described in the protocol.

10.6.2 Substantial amendment

Significant changes require a substantial amendment. Significant changes include but are not limited to: new data affecting the safety of subjects, change of the objectives/endpoints of the study,

eligibility criteria, dose regimen, study assessments/procedures, treatment or study duration, with or without the need to modify the core Subject Information and Informed Consent Form.

Substantial amendments are to be approved by the appropriate EC and the CA will need to provide a 'no grounds for non-acceptance' notification prior to the implementation of the substantial amendment.

Urgent amendment

An urgent amendment might become necessary to preserve the safety of the subjects included in the study. The requirements for approval should in no way prevent any immediate action being taken by the investigators in the best interests of the subjects. Therefore, if deemed necessary, an investigator can implement an immediate change to the protocol for safety reasons. This means that, exceptionally, the implementation of urgent amendments will occur before submission to and approval by the EC(s) and CA.

10.7 End of study report

The investigator will notify the EC and the CA of the end of the study within a period of 90 days. The end of the study is defined as the last subject's last visit.

In case the study is ended prematurely, the investigator will notify the EC and the CA within 15 days, including the reasons for the premature termination.

Within one year after the end of the study, the investigator will submit a final study report with the results of the study, including any publications/abstracts of the study, to the EC and the CA. The principal investigator will be the signatories for the study report.

10.8 Public disclosure and publication policy

In accordance with standard editorial and ethical practice, the results of the study will be published. The authorship guidelines of the Vancouver Protocol[3] will be followed regarding co-authorship.

The principal investigator will have the opportunity to review the analysis of the data and to discuss the interpretation of the study results prior to publication.

[3] http://www.icmje.org/

11 STRUCTURED RISK ANALYSIS

Please refer to the respective Summary of Product Characteristics (SmPC, see appendix 1) and the European Public Assessment Report (EPAR, see appendix 3).

12 REFERENCES

References

1. Dr.Dick Marty, Mr.Peter Nicholson, Prof.Dr.Ulrich Haas. Report to the president of the Union Cycliste Internationale. *Cycling Independent Reform Commision* 2015.

2. Nimesh SA Patel, Massimo Collino, Muhammad M Yaqoob, Christoph Thiemermann. Erythropoietin in the intensive care unit: beyond treatment of anemia. *Annals of Intensive Care* 2011; 1: 1-9.

3. [online] Available at http://www.wada.-ama.org/e. *World Anti-Doping Agency* 2015.

4. Heuberger JA, Cohen Tervaert JM, Schepers FM, Vliegenthart AD, Rotmans JI, Daniels JM, Burggraaf J, Cohen AF. Erythropoietin doping in cycling: lack of evidence for efficacy and a negative risk-benefit. *Br J Clin Pharmacol* 2013; 75: 1406-1421.

5. Jeukendrup AE, Craig NP, Hawley JA. The bioenergetics of World Class Cycling. *J Sci Med Sport.* 2000; 3: 414-433.

6. Connes P, Perrey S, Varray A, Prefaut C, Caillaud C. Faster oxygen uptake kinetics at the onset of submaximal cycling exercise following 4 weeks recombinant human erythropoietin (r-HuEPO) treatment. *Pflugers.Arch.* 2003; 447: 231-238.

7. Lucia A, Hoyos J, Perez M, Santalla A, Chicharro JL. Inverse relationship between VO2max and economy/efficiency in world-class cyclists. *Med Sci.Sports Exerc.* 2002; 34: 2079-2084.

8. Norio Suzuki. Erythropoietin Gene Expression: Developmental-Stage Specificity, Cell-Type Specificity, and Hypoxia Inducibility. *Tohoku J.Exp.Med.* 2015; 235: 233-240.

9. Lucia A, Hoyos J, Santalla A, Perez M, Chicharro JL. Curvilinear VO(2):power output relationship in a ramp test in professional cyclists: possible association with blood hemoglobin concentration. *Jpn J Physiol.* 2002; 52: 95-103.

10. Heinicke K, Wolfarth B, Winchenbach P, Biermann B, Schmid A, Huber G, Friedmann B, Schmidt W. Blood volume and hemoglobin mass in elite athletes of different disciplines. *Int J Sports Med* 2001; 22: 504-512.

11. Henny H.Billett. Hemoglobin and hematocrit.In: *Clinical Methods: The History, Physical, and Laboratory Examinations. 3rd edition.* 1990: 718-719.

12. Online available at http://www.blood.ca/en/blood/hemoglobin. *Canadian Blood Services* 2015.

13. Wilkerson DP, Rittweger J, Berger NJ, Naish PF, Jones AM. Influence of recombinant human erythropoietin treatment on pulmonary O2 uptake kinetics during exercise in humans. *J Physiol.* 2005; 568: 639-652.

14. Thomsen JJ, Rentsch RL, Robach P, Calbet JA, Boushel R, Rasmussen P, Juel C, Lundby C. Prolonged administration of recombinant human erythropoietin increases submaximal performance more than maximal aerobic capacity. *Eur.J Appl.Physiol.* 2007; 101: 481-486.

15. Lundby C, Achman-Andersen NJ, Thomsen JJ, Norgaard AM, Robach P. Testing for recombinant human erythropoietin in urine: problems associated with current anti-doping testing. *J Appl.Physiol.* 2008; 105: 417-419.

16. Birkeland KI, Stray-Gundersen J, Hemmersbach P, Hallen J, Haug E, Bahr R. Effect of rhEPO administration on serum levels of sTfR and cycling performance. *Med Sci.Sports.Exerc.* 2000; 32: 1238-1243.

17. Ralph B.Dell, Steve Holleran, Rajasekhar Ramakrishnan. Sample Size Determination. *ILAR journal* 2002; 43: 207-213.

18. Gore CJ, Parisotto R, Ashenden MJ, Stray-Gundersen J, Sharpe K, Hopkins WG, Emslie KR, Howe C, Trout GJ, Kazlauskas R, Hahn AG. Second-generation blood tests to detect erythropoietin abuse by athletes. *Haematologica* 2003; 88: 333-343.

19. Sottas PE, Robinson N, Giraud S, Taroni F, Kamber M, Mangin P, Saugy M. Statistical Classification of Abnormal Blood Profiles in Athletes. *The International Journal of Biostatistics* 2006; 2: 3.

13 APPENDIX 1 – SUMMARY OF PRODUCT CHARACTERISTICS – ENGLISH VERSION

14 APPENDIX 2 – SUMMARY OF PRODUCT CHARACTERISTICS – DUTCH VERSION

15 APPENDIX 3 – EUROPEAN PUBLIC ASSESSMENT REPORT

16 APPENDIX 4 – SCREENING: QUESTIONNAIRE SPORT ACTIVITIES

Sport activities in the past 3 years:

	Type of sport	Times a week	Distance and duration per training/competition	Mean velocity	Since when
1					
2					
3					
4					
5					

Sport activities in the past 6 weeks:

	Type of sport	Times a week	Distance and duration per training/competition	Mean velocity	Since when
1					
2					
3					
4					
5					

Dutch:

Sport activiteiten in de afgelopen 3 jaar:

	Type sport	Aantal keer per week	Afstand en duur van training/competitie	Gemiddelde snelheid (km/u)	Sinds wanneer
1					
2					
3					
4					
5					

Sport activiteiten in de afgelopen 6 weken:

	Type sport	Aantal keer per week	Afstand en duur van training/competitie	Gemiddelde snelheid (km/u)	Sinds wanneer
1					
2					
3					
4					
5					

17 APPENDIX 5 – EXERCISE TEST

During this study the exercise tests will be performed on a cycle ergometer.
The subjects will cycle at a self-selected pedal rate (between 70 and 90 rpm) and this pedal rate along with the saddle and handlebar heights will be recorded and reproduced in subsequent tests.

All subjects will breathe through a facemask that is connected to an oxymeter to collect inspired and expired gasses for analyzing oxygen consumption, carbon dioxide production, minute ventilation, tidal volume and breathing frequency. Before each test, the oxymeter will be calibrated to ensure valid measurements. Blood pressure and heart rate will also be monitored during the tests. The blood pressure and heart rate measuring devices, cycle ergometer and oxymeter will be connected to a computer to measure and analyse all data.

18.1 Ramp exercise test

Protocol
A ramp protocol will be followed until exhaustion, which will take approximately 1 hour. The details of the ramp exercise test will be described in the exercise test manual.

18.2 Time to exhaustion test

Protocol
This exercise test will be performed according to a protocol looking at performance at submaximal intensity. It will last approximately 1 hour. The details of time to exhaustion test will be described in the exercise test manual.

18.3 Statistics

All data will be presented as means ± SD (or standard error). First, a two-way factor (group versus respectively V_{O2}, V_{O2}/kg, %V_{O2max}, W and W/kg) ANOVA with repeated measures will be performed to determine if an interactive effect exists between both groups and mentioned parameters. These parameters will be analyzed at VT1, VT2 and maximal intensity. The ANOVA will be conducted to determine whether the factor group will significantly influence these physiological parameters. When a significant F-ratio is detected, comparisons for unpaired data will be performed (Student’s t-tests) to locate the differences (2-tailed). Maximal lactate values between both groups will also be compared using an unpaired Student’s t-test. V_{O2} at the ventilatory and lactate thresholds will be used to compare both thresholds by using a paired Student’s t-test. Statistical significance will be set at $P < 0.05$.

18 APPENDIX 6 – COMPETITION

Subjects will climb the Mont Ventoux in an open course via Bédoin after a 150km closed course. A blood sample of each subject will be collected in Bédoin (at the end of the closed course) and at the finish. A power meter will be incorporated into each bike.

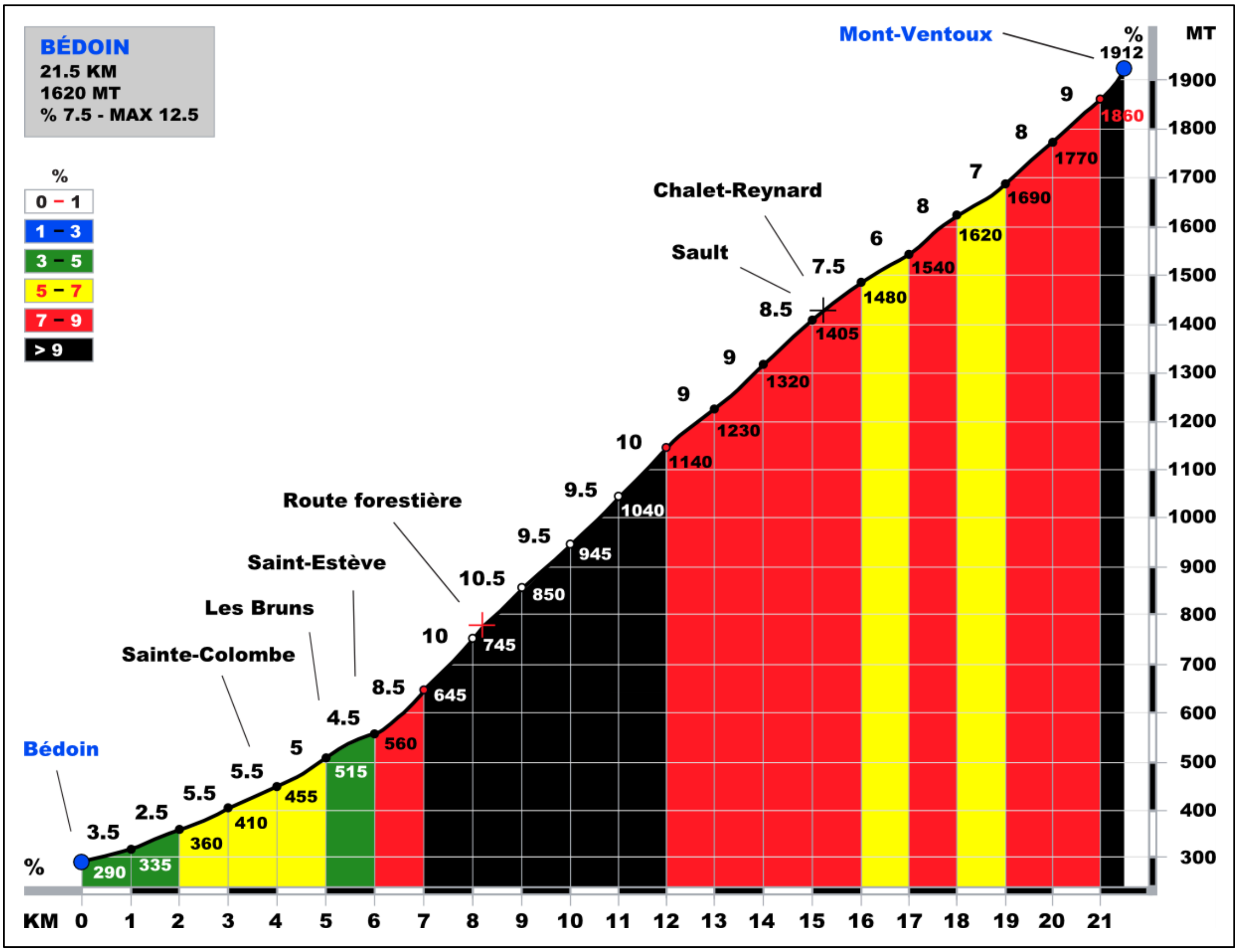

Figure 2. Detailed description of the Mont Ventoux climb via Bédoin.[1]

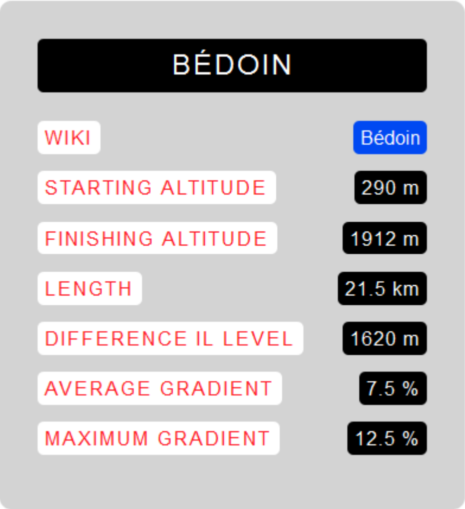

Figure 3. Characteristics of the Mont Ventoux climb via Bédoin.[1]

References

1. http://www.clubcinglesventoux.org/en/roads/1-bedoin.html

19 APPENDIX 7 – URINE DOPING CONTROL PROCEDURE

Urine collection for doping detection will be done at predetermined moments as specified in the Schedule of Assessments. For each sample, a minimum of 50 mL urine will be collected, which will be stored at -20 degrees Celcius or below before shipment to the Doping Lab.

The doping lab will process the samples in a blind fashion as per the WADA Technical document published online at https://www.wada-ama.org/en/resources/science-medicine/td2014-epo.

Samples will be analysed using two methods, being the SAR-PAGE and Isoelectrofocusing (IEF) as described in this technical document.
